# Genomic and epidemiological characterisation of a dengue virus outbreak among blood donors in Brazil

**DOI:** 10.1038/s41598-017-15152-8

**Published:** 2017-11-09

**Authors:** Nuno R. Faria, Antonio Charlys da Costa, José Lourenço, Paula Loureiro, Maria Esther Lopes, Roberto Ribeiro, Cecilia Salete Alencar, Moritz U. G. Kraemer, Christian J. Villabona-Arenas, Chieh-Hsi Wu, Julien Thézé, Kamran Khan, Shannon E. Brent, Camila Romano, Eric Delwart, Brian Custer, Michael P. Busch, Oliver G. Pybus, Ester C. Sabino, NHLBI Recipient Epidemiology and Donor Evaluation Study-III (REDS-III), Cesar de Almeida Neto, Alfredo Mendrone-Junior, Anna Bárbara Carneiro-Proietti, Divaldo de Almeida Sampaio, Clarisse Lobo, Ligia Capuani, João Eduardo Ferreira, Marcio Oikawa, Pedro Losco Takecian, Cláudia Di Lorenzo Oliveira, Shannon Kelly, Thelma T. Gonçalez, Donald Brambilla, Christopher McClure, Simone A. Glynn

**Affiliations:** 2 Instituto de Medicina Tropical, Universidade de São Paulo, São Paulo, Brazil; 3 LIM46, Departamento de Moléstias Infecciosas e Parasitárias, Faculdade de Medicina, Universidade de São Paulo, São Paulo, Brazil; 4 Faculdade de Ciências Médicas, Fundação Hemope, Recife, Brazil; 5 Fundação Hemório, Rio de Janeiro, Brazil; 11 Blood Systems Research Institute, San Francisco, California USA; 13 Fundação Pró-Sangue Hemocentro de São Paulo, São Paulo, Brazil; 14 Fundação Hemominas, Belo Horizonte, Minas Gerais, Brazil; 15 Universidade Universidade Federal de São João del-Rei, São João del-Rei, Minas Gerais, Brazil; 16 RTI international, Rockville, Maryland USA; 17 National Heart, Lung, and Blood Institute, National Institutes of Health, Bethesda, Maryland USA; 1 Department of Zoology, University of Oxford, Oxford, United Kingdom; 6 Laboratório de Investigação Médica LIM03, Hospital das Clínicas, Sao Paulo, Brazil; 7 Institut de Recherche pour le Développement, Université de Montpellier, Montpellier, France; 8 Department of Statistics, University of Oxford, Oxford, United Kingdom; 9 Li Ka Shing Knowledge Institute, St. Michael’s Hospital, Toronto, Ontario Canada; 10 Division of Infectious Diseases, University of Toronto, Toronto, Ontario Canada; 12 University of California San Francisco, San Francisco, California USA

## Abstract

Outbreaks caused by Dengue, Zika and Chikungunya viruses can spread rapidly in immunologically naïve populations. By analysing 92 newly generated viral genome sequences from blood donors and recipients, we assess the dynamics of dengue virus serotype 4 during the 2012 outbreak in Rio de Janeiro. Phylogenetic analysis indicates that the outbreak was caused by genotype II, although two isolates of genotype I were also detected for the first time in Rio de Janeiro. Evolutionary analysis and modelling estimates are congruent, indicating a reproduction number above 1 between January and June, and at least two thirds of infections being unnoticed. Modelling analysis suggests that viral transmission started in early January, which is consistent with multiple introductions, most likely from the northern states of Brazil, and with an increase in within-country air travel to Rio de Janeiro. The combination of genetic and epidemiological data from blood donor banks may be useful to anticipate epidemic spread of arboviruses.

## Introduction

Dengue virus (DENV) is one of the most important vector-borne diseases worldwide, causing an estimated 390 million infections annually in over 100 countries^1^. DENV belongs to the *Flavivirus* genus and is classified into four genetically and antigenically distinct serotypes, DENV1-4^2,3^. The geographic range of DENV types has dramatically increased in recent decades^4^, fueled by the expansion of its main vector species, the *Aedes aegypti* mosquito, human population growth, international travel and trade, and increasing urbanization in the tropics and subtropics^5,6^.

Brazil has reported the highest number of dengue cases worldwide, with >7.3 million notified infections between 2000–2013^7^, and transmission occurring in most of the country^8^. Current estimates of DENV burden in Brazil may however be largely underestimated; recent work suggests that actual number of cases may be 12- to 17-fold higher than those captured by passive case reporting implemented in the country^9^. In Latin America DENV disease affects mostly adults and manifests as primary dengue fever (DF). However, the clinical and epidemiological profile of DENV in Brazil has been shifting in recent years. As a result of the increased co-circulation of different DENV serotypes in the country, DENV infection is now becoming associated with more severe cases in younger populations^10^. In addition, Brazil has experienced recurrent severe DENV outbreaks of increasing magnitude over the past three decades, with particularly high incidence peaks in 2002, 2008, 2010, and 2012^11^.

After more than two decades of absence from Brazil, DENV4 has been re-introduced at least six times into the northern part of the country until 2011^12^. This region is characterized by a high suitability for *Aedes* mosquitoes^13,14^ but has the lowest human population density in Brazil and comparatively poor transportation connections to the rest of the country. Although most of these DENV4 introductions resulted in short-lived transmission chains, in 2012–2013 DENV4 spread beyond the northern region and caused large urban outbreaks in central and southern Brazil^15^. Today, the four DENV serotypes co-circulate in an increasing number of Brazilian regions^4,16–18^.

Despite the morbidity and mortality caused by DENV infection, there is limited information about what drives the transmission potential and spatial spread of novel DENV lineages, since most epidemiological studies rely on clinical case reports, which rarely discriminate infection by DENV serotype and are sometimes confounded by infections caused by other arthropod-borne viruses, for example Chikungunya, Zika, Oropouche or Mayaro virus. Further, current investigations of DENV dynamics often do not fully exploit the information contained in genome sequences about virus introduction, invasion and persistence and report only cross-sectional case data, whilst longitudinal data are needed for detailed investigation of viral population dynamics^19^. Blood donor screening, combined with retrospective analysis of circulating virus strains represents a valuable alternative approach for epidemic characterisation and the early detection of newly emergent flaviviruses^20–23^.

**Figure 1:**
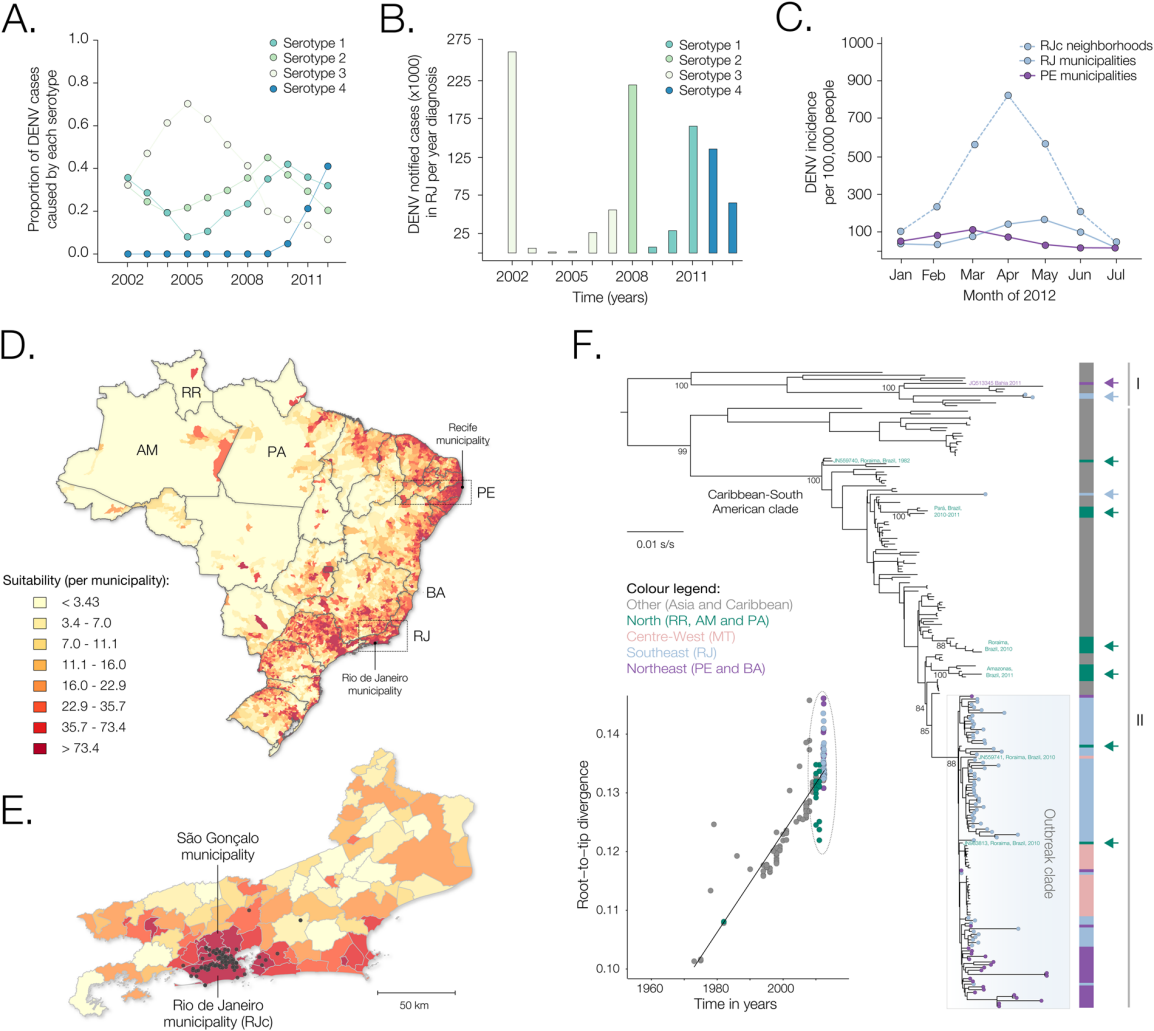
Patterns of DENV4 transmission in Rio de Janeiro and Pernambuco, Brazil. (**A)** Proportion of annual dengue serotype-specific detections in Brazil obtained from SINAN reports. (**B**) Total number of dengue notified cases for each serotype in Rio de Janeiro (RJ) between 2002 and 2013^74^. The same colour scheme was used in panels A and B. (**C**) Notified DENV incidence in RJ state, in RJ municipality/city (RJc) neighborhoods, and in Pernambuco (PE) municipalities, as obtained from SINAN reports. (**D**) Cartography of Brazil showing suitability for *Aedes*-borne viruses, *i*.*e*. presence of *Ae*. *aegypti* described in^13^ weighted by population density (aggregated per municipality) in each 5,596 Brazilian municipalities for the same year^29^. Most suitable areas are located in northeastern and southeastern areas of the country. (**E**) Zoomed map of the RJ state where black circles indicate patient’s most likely place of infection (residence) (http://www.worldpop.org.uk/). (**F**) Mid-point rooted ML phylogenetic tree using 244 genome sequences from DENV4 sampled worldwide. Coloured circles at the tips of phylogenetic branches represent sequences reported in this study, and gave been coloured according to location of sampling (as shown on the left of the phylogeny). Sylvatic strains (Accession numbers: EF457906, JF262779 and JF262780, all from Malaysia) were removed for clarity. A fully annotated ML tree is show as Fig. S1, and the corresponding molecular clock tree is shown as Fig. S2. The blue box highlights the DENV4-II outbreak clade in Brazil studied here (see also Fig. 2). Green arrows on the right denote secondary introductions of DENV4 in the Northern region of Brazil (RR: Roraima, PA: Pará and AM: Amazonas states) that resulted in little or no onward transmission in the country. Blue and purple arrows denote, respectively, secondary introductions of non-outbreak strains in RJ and PE. Colour code for the bars right-sided to the phylogeny is shown on the left of the phylogeny. Numbers below key nodes indicate bootstrap support. Left to the tree, a linear regression analysis between divergence and sampling times for the global tree depicted in panel F is shown. Maps were created using the ggplot2 package in “R version 3.3.3”.

The first report of DENV4 genotype II (DENV4-II) in Brazil was in 1981–1982 in Boavista, the capital city of the northernmost state of Roraima^24^. After a limited outbreak there, DENV4 was absent from the country until 2005–2007, when it was detected in the northern Amazon state that neighbours Roraima^24^. In 2010 DENV4 was found again in other northern states (Fig. 1A). However, it was only in 2012 that DENV4-II caused large outbreaks in more densely populated states of Brazil, such as Rio de Janeiro (RJ) and Pernambuco (PE) (Fig. 1B). The municipality of Rio de Janeiro is an important tourism destination, with well-connected air, road and railway networks and one of the largest harbours in the Americas. Local *Aedes spp*. populations are highly susceptible to DENV^25^ and previous studies suggest that Rio de Janeiro has played a key role in the national dissemination of several dengue serotypes, including DENV1 in 1986^26^, DENV2 in 1990^27^ and DENV3 in late 2000^28^.

In order to characterize and understand the epidemic behaviour of the lineage that caused the 2012 outbreak of DENV4 we undertook retrospective molecular surveillance of the largest Brazilian study of transfusion-transmitted DENV^22^. This included a total of 40,278 blood donors and recipients in the capital cities of RJ and PE states, namely in Rio de Janeiro city (RJc), and Recife. In our study, individuals were recruited between January and July 2012 and a mean DENV RNA + prevalence (per unit) of 0.51% was found in Rio de Janeiro, and 0.80% in Recife^22^. From these RNA + samples, we obtained a total of 92 complete or near-complete DENV4 genomes using next generation sequencing. We then combined evolutionary analysis with ento-epidemiological modelling to investigate the introduction, spread and impact of environmental factors on the 2012 DENV4-II outbreak. Our study demonstrates the potential of blood bank screening for understanding the epidemiology and molecular evolution of DENV and other arboviruses currently circulating in Brazil.

## Results

A total of 147 DENV RNA + samples were obtained and submitted to amplification of whole genome, of those 92 were PCR + for at least one of the 5 fragments. The length of the consensus genome sequences obtained varied between 1,215 and 10,066 nt in length, with a median of 8,752 nt, of which 42 were whole genomes (for details see Table S1). From the remaining 57 DENV RNA + samples that failed to amplify, viral load was available for 42, and only 2 samples had a viral load > 10^3^ copies/mL (1,116 and 3,100 copies/mL). As expected, viral loads were positively correlated with the length of the consensus genome obtained (Pearson’s correlation, adjusted r^2^ = 0.62, *p*-value < 0.0001).

There was a strong correlation between the total number of DENV RNA + samples in blood bank donors and recipients in each month and the number of notified dengue infections in RJ municipalities during that month (Pearson’s correlation for RJ municipalities: adjusted r^2^ = 0.97, *p*-value = 0.0002) and in RJc neighbourhoods (adjusted r^2^ = 0.86, *p*-value = 0.011). Further, we found a moderate but significant spatial correlation between the number of cases in each municipality in RJ or RE and the estimated human population density in that municipality (Spearman’s rank correlation, $\rho$ = 0.72; p-value < 0.001; human densities were obtained from high-resolution gridded datasets for Brazil^29^, Fig. 1D,E). This suggests that the demographics of the blood donor-recipient cohort analysed here was roughly representative of the population under study.

To place our newly generated DENV genomes into a global evolutionary context, we estimated maximum likelihood (ML) and Bayesian molecular clock phylogenies from an alignment comprising a total of 218 DENV4 genomes sampled worldwide sampled from 1972–2013, including the 92 novel Brazilian genomes reported here (Fig. 1F); a fully annotated phylogeny is shown respectively as Fig. S1. Both the ML and Bayesian phylogenies show that 98% (88 of 90) of the novel Brazilian DENV isolates fall within a single well-supported monophyletic clade (maximum likelihood bootstrap score, BS, = 87; Bayesian posterior probability, PP, = 0.99). This clade is closely related to viruses from Venezuela and from the northern Brazil region that contain the capital cities of Roraima (RR) and Amazonas (AM), that have a high suitability for *Ae*. *aegypti* and high human population density (Fig. 1D,F).

There was a strong correlation between the genetic distance of each sequence to the root of the global DENV4 phylogeny and the date of sequence sampling for the global dataset (Fig. 1F inset, r^2^ = 0.76). Despite this, the relaxed molecular clock model exhibited a high among-lineage coefficient of variation (1.74; 95% Bayesian credible interval = 1.38–2.14), indicating significant rate heterogeneity among branches. Such high among-lineage rate variability is not unexpected for flavivirus phylogenies that comprise genomes that have been longitudinally sampled over 40 years^19,30,31^. The estimated genomic evolutionary rate for the global DENV4 dataset was 1.36 × 10^−3^ substitutions per site per year, s/s/y (95% Bayesian credible interval = 1.17–1.53 × 10^−3^ s/s/y).

Our data also reveals for the first time the circulation of DENV4 genotype I (DENV4-I) in Rio de Janeiro. The two DENV4-I isolates from RJ cluster together with maximum statistical support (BS = 100, PP = 1.00) and are related to isolates sampled in Southeast Asia in 2002–2011 (Fig. 1F). In addition, these two isolates are polyphyletic with respect to a single DENV4-I isolate previously detected in Bahia state in 2011^12^. One DENV4-I isolate was also detected in 2012 in the harbour city of Santos, São Paulo state (AC da Costa, personal communication). Together, these data suggest that DENV4-I appears to have been introduced to Brazil from Southeast Asia on at least two separate occasions.

The time of the common ancestor of the outbreak clade was estimated to be February 2010 (95% Bayesian credible intervals, BCI: April 2009 to September 2010, Fig. 2A). In February 2011, DENV4 was confirmed for the first time in the northeast of Brazil (states of Bahia, Pernambuco and Piauí) and in May 2011 the virus was confirmed in the southeast (São Paulo and Rio de Janeiro)^32,33^, but fewer than 100 cases were detected in total in these locations in 2011.

**Figure 2:**
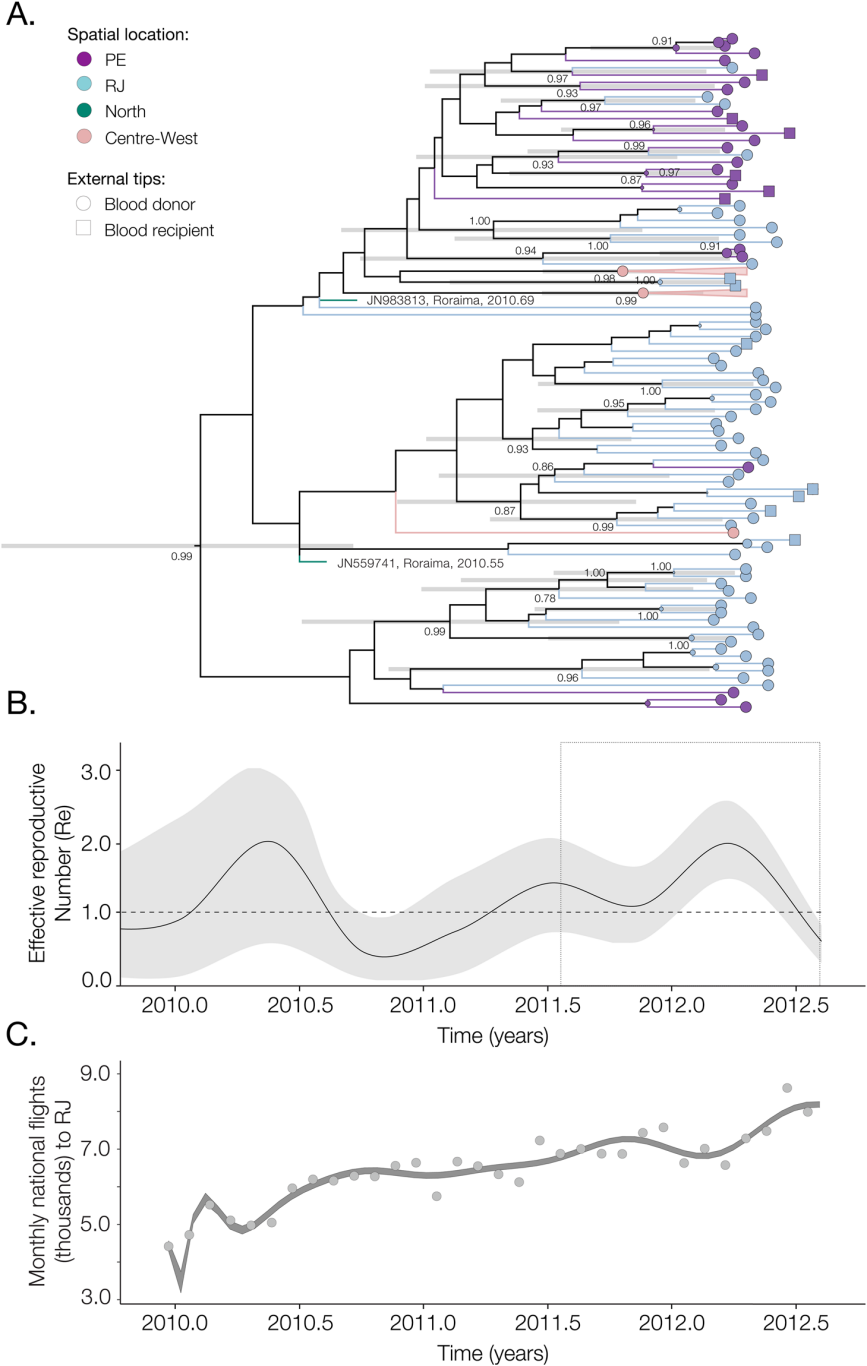
Seasonal dynamics of the 2012 DENV4 outbreak in Brazil. (**A**) Time-calibrated phylogenetic tree of the outbreak clade. This represents a subset of the global phylogenetic tree shown in Fig. 1. Circles and squares at the external branches represent isolates from donors and recipients respectively, and colours represent cohort locations. Horizontal bars represent the 95% credible intervals of all divergences that are supported by posterior probabilities above 0.9. (**B**) Temporal fluctuation of the effective reproduction number (R_e_) of the outbreak clade estimated using the Bayesian birth-death approach (see details in Materials and Methods). (**C**) Polynomial fitting of the aggregated number of passengers arriving on national flights (dark grey points) to RJ between January 2010 and July 2012.

Crucially, DENV4 had never been confirmed in Rio de Janeiro municipality before mid-May 2011, raising the following question: where did the outbreak lineage reside before its earliest detection in the city? One hypothesis is that the most recent common ancestor of the outbreak strains in Rio descends from a single lineage that circulated undetected in the city prior to the ignition of the outbreak (*scenario A*). Alternatively, it is possible that the outbreak in Rio de Janeiro has multiple ancestors, each of them originating from a pool of unsampled diversity outside of Rio. Likely locations of this source population are the urban areas of the Brazilian or Venezuelan Amazon with high suitability for DENV transmission (*Scenario B*). Consistent with this we observe that (i) phylogenetic analyses place the ancestors of past Brazilian DENV4-II outbreaks in Venezuela (Fig. 1F and Fig. S1), which is connected by the Branco and Amazon rivers to Boavista (Roraima state) and Manaus (Amazonas state), and (ii) two isolates sampled in mid-2010 in the city of Roraima are consistently placed amongst the early lineages of the outbreak clade (Fig. 1F and Fig. 2A).

We next sought to investigate why and how the outbreak in Rio de Janeiro ignited only in January 2012, and not before. Due to the extensive genetic sampling of the outbreak, we were able to estimate temporal fluctuations in its effective reproduction number, *R*_e_, using the birth-death skyline (BDSKY) model, based on a phylogenetic birth-death process^34^. *R*_*e*_ is defined as the average number of potential secondary infections from an infected individual in the presence of herd-immunity at each point in time during the outbreak. *R*_*e*_ > 1 indicates increasing number of cases, and a potential epidemic, while *R*_*e*_ < 1 indicates that the number of cases decreases with each generation, and if the decrease is maintained the epidemic with be eliminated. *R*_*e*_ equals the basic reproduction number, *R*_0,_ at the start of an epidemic when the host population is completely susceptible.

The BDSKY results are shown in Fig. 2B. Although the BDSKY uncertainty intervals are larger in the past, the analysis shows seasonal variations that are consistent with epidemiological information (Fig. 1C). *R*_e_ started to increase around December 2011 and peaked around mid-March 2012, at a value of *R*_*e*_ = 1.9 (95% BIC: 1.5–2.6) (Fig. 2B). Around mid-June 2012, estimated *R*_*e*_ declines to <1, coinciding with the observed end of the epidemic. More generally, the BDSKY suggests a rise during the first 3 months of 2012 followed by a fall in *R*_e_ < 1 in the second quarter of the year.

The detection of seasonal fluctuations across multiple years is encouraging given that almost all sequences used in analysis were sampled in the first half of 2012. Using the BDSKY approach we also estimated the generation time, i.e. the period between two infections mediated by the vectors, to be 21 days (95% BCI: 11 to 34 days). The proportion of cases that were sampled was estimated to be 3.42 × 10^−5^ (95% BCI: 2.12 × 10^−8^ to 1.22 × 10^−4^). If we consider human population sizes of the three sampled federal states from which genome data was analysed (RJ, PE and MS), this corresponds to an infected population of at least 940,000 individuals until the end of July 2012, which is around the triple of the number of official cases notified during this period in Brazil (Fig. 1b). The phylogeny is significantly structured by geographic location (federal state) of sampling (Association index = 0.43; bootstrap significance = 1.00), but this spatial structure does not appear to impact our BDSKY estimates for 2012 since the model captures well the observed oscillations in notified cases (Fig. 1C).

To investigate whether the ignition of the DENV4 outbreak in Rio de Janeiro was linked with an increase in the number of travelers from other destinations within Brazil, we collated data on the number of passengers arriving on commercial flights into Rio de Janeiro from all others airports across Brazil between January 2010 and December 2012. These data were obtained from the International Air Transportation Association, which collects data on an estimated 90% of all passenger trips worldwide. We find that between 2010 and July 2012, the number of passengers per month to Rio de Janeiro nearly doubled, increasing on average 77% per year during this period (Fig. 2C).

**Figure 3:**
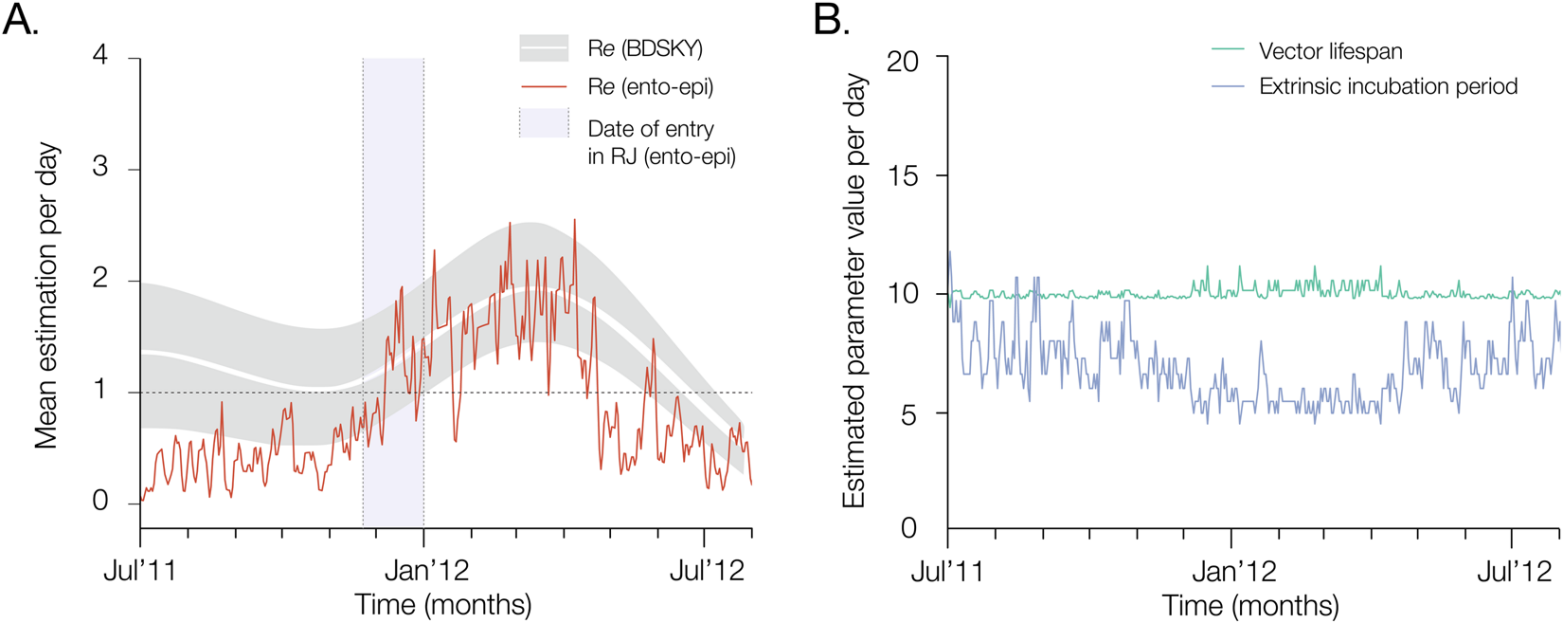
Epidemiological and entomological dynamics of a dengue virus outbreak. (**A**) The red solid line corresponds to daily *R*_*e*_ estimates obtained using the ento-epidemiological model for the period July 2011 to July 2012 in Rio de Janeiro state, Brazil. In grey are the 95% credible intervals obtained using the BDSKY model as shown in Fig. 2B, with the white solid line representing the mean estimates of *R*_*e*_ through time. In panel B, the green line corresponds to the lifespan of *Aedes* mosquitoes, while the purple line corresponds to the vector’s extrinsic incubation period. The estimated date of entry of DENV4 in Rio de Janeiro (blue box in panel A) was November to December 2011.

Finally, we used a susceptible-exposed-infected-recovered (SEIR) mathematical model to explore in detail the ecological conditions and vector transmission dynamics underlying the 2012 outbreak, whilst explicitly accounting for temperature variation as a major determinant of vector capacity^35^ (see Methods). A time series of incidence during the outbreak was compiled from the DENV4 RNA + weekly cases in Rio de Janeiro and fitted to the model. Figure 3 shows the daily estimates of key epidemiological parameters from the SEIR model. Notably, the initial increase and peak in *R*_*e*_ in 2012 observed from genetic data using the BDSKY approach (Fig. 2B) matches the estimates of *R*_*e*_ from the ento-epidemiological SEIR model (Fig. 3A). However, the lower *R*_*e*_ until end-2011 estimated from the genetic data may reflect the absence of pre-outbreak dengue isolates (Fig. 1B). We obtain a similar trend using the ento-epidemiological SEIR model; since the latter does not depend on pre-outbreak case counts, these findings support the notion that mosquito and temperature factors were insufficient for outbreak transmission between mid-2011 and early 2012. According to the SEIR model, the date of the first human case of the DENV4-II outbreak in Rio de Janeiro occurred around Nov-Dec 2011. This timing is consistent with our molecular clock estimates and with multiple concurrent introductions to Rio de Janeiro from northern Brazil with high suitability for DENV endemicity (*Scenario B*).

Besides capturing the dynamics of the DENV4 outbreak (Fig. 3A), the ento-epidemiological SEIR model recovers several other parameters such as the life expectancy and extrinsic incubation period of the mosquito vector (Fig. 3B). In line with previous observations from field mosquitoes in Rio de Janeiro^36,37^, we estimated the extrinsic incubation period of *Aedes* (i.e. time necessary for the virus to be detected in saliva and ready for transmission after being ingested with the blood meal) to be around 7 days (95% CI: 4.4–10 days), and its life span around 10.5 days (95% CI: 10.1–12.3).

## Discussion

In this study, we characterized the dynamics of a large urban dengue virus outbreak in Brazil. By combining genetic and epidemiological models applied to blood bank surveillance data, we show that the establishment of the DENV4-II outbreak in Brazil was contingent upon the introduction of new strains to large population centres during a time window of high transmission potential (R_e_ > 1). We obtained similar epidemic trends and parameter estimates using two fundamentally different approaches based on independent data sources, a birth-death phylodynamic model and an ento-epidemiological SEIR model. Notably, the estimated peak of epidemic transmission in March 2012 (R_e_ ∼ 2) coincides with the observed peak in DENV detection in blood-donor banks, highlighting the potential of blood bank screening for surveillance and early outbreak detection. More generally, the urban and peri-urban areas of residence of blood donor/recipient population under study may be highly suitable for arboviral outbreaks, as measured by *Aedes* suitability weighted by human population density (Fig. 1E).

Our study emphasises that sequence data can support and corroborate epidemiological data when the former is weak, absent, or affected by under-reporting. The current DENV surveillance system in Brazil is based on passive surveillance, with clinical testing through antigen detection assays. There is often serological cross-reactivity, not only among different DENV lineages but also with other circulating Flaviviruses^38–40^. Approximately 213,000 dengue cases were notified to the national surveillance system in RJ and PE during 2012. Consistent with previous seroincidence studies^22,41^, we estimate at least a 3.4-fold greater number of dengue infections compared to those that resulted in reported disease. The approach described here is directly applicable to other arboviruses that spread via the same *Aedes* vector such as Chikungunya, Zika and urban yellow fever virus.

Temperature-based models and entomological surveys have shown that the Amazon region of Brazil is highly suitable for year-round DENV transmission^42–44^. Since the clade responsible for the 2012 outbreak seems to descend from viral strains circulating in the Amazon region, the most parsimonious explanation for the absence of notified cases in urban centres under study until 2012 (Fig. 1B) is that DENV4-II circulated undetected in the northern tropical areas of South America. A similar pattern has been observed for specific genotypes of DENV types 1, 2 and 3^30^. This suggests a sink-source dynamic for DENV in Brazil, with locations in the northern region acting as a DENV reservoir, or as a stepping-stone location for virus spread from countries outside Brazil to the rest of Brazil. Further, the fact that *Re* is only >1 for part of the year in Rio, as shown by the SEIR model and the BDSKY, indicates that Rio consistently receives new DENV virus lineages. In other words, transmission chains within Rio are unlikely to be sustained across multiple seasons and will therefore require re-seeding from a source location; this re-seeding is a shaped by local vector ecology and human mobility^30,45^. While the incorporation of vector species dynamics into a viral evolutionary framework remains challenging^46^, the modeling approach employed here could be extended to capture not only multi-vector dynamics but to generate daily estimates of key parameters using real-time temperature fluctuations.

Human population density in Brazil is highest in the eastern and southern coastal urban centres^29^, in climatic areas where the risk of DENV establishment after its introduction is usually limited to a particular yearly window. For Rio de Janeiro, which hosted the Olympic and Paralympic Games in August and September 2016, our results suggest that the risk that an introduced arbovirus lineage (against which there is little herd immunity) will generate a sustained chain of transmission is highest between November and December, which coincides with the peak in annual passengers arriving on commercial flights into Rio de Janeiro (Fig. 2). Thus, whilst introduction of new DENV lineage or other arboviruses to Rio de Janeiro is a continual process, there was little risk that introductions would ignite large local outbreaks within Rio as a result of these popular sporting events.

Our study also provides the first report of an introduction of DENV4-I in Rio de Janeiro. A single genome isolate of DENV4-I had been previously collected in March 2011 in Bahia^12^, but the pair of sequences in Rio de Janeiro identified here reveals a second independent introduction of this genotype into Brazil, and there is some evidence that DENV4-I exhibited limited circulation in the Amazonas state in 2008^47^.

Our estimates of viral and vector parameters obtained using mathematical modelling are consistent with estimates previously published for DENV and *Aedes* vectors. Namely, our within-season (years 2009–2012, months December to April) estimates of R_e_ for DENV4-II are around 2.2 (0.62–4.3), in line with previous estimates^35,48–50^. Also, we found temperature variation to be a key driver of parameters dictating vector capacity, consistent with other studies^35,42^; estimated means of vector lifespan and EIP were well within that expected for the tropical wet and dry climate of Rio de Janeiro^36,37^. Both the genetic and time series data captured well seasonal oscillations between January and July 2012. However, estimates of transmission potential (R_e_) under the two approaches varied considerably before January 2012. Both models are likely to lose predictive power prior to that date due to the absence of genetic or case count data to be fitted.

Genomic surveillance of blood banks is particularly useful for viral epidemics that may spread undetected in immunologically naïve populations. For example, the explosive spread of Zika virus in Brazil went undetected for at least one year^56,75^ before first being reported in March 2015^51^. Further, there is the possibility that West Nile virus (recently found in humans in Piauí state^52^), the ECSA genotype of Chikungunya virus spreading in the northeast of Brazil^53,54^, or the Japanese encephalitis virus currently circulating in South Asia may cause future arboviral epidemics in Brazil. There is a need for continuous pathogen surveillance to protect blood supplies and reduce the risk of transfusion-associated transmission. We expect identical or near identical viruses to be transmitted from the blood donor-recipient pairs. Unfortunately, of the six donor-recipient DENV RNA + sample pairs, we could not obtain sequences for both donor and recipient to inspect whether these were phylogenetically linked.

Although we investigated the epidemic of a DENV4 urban outbreak, we did not evaluate the impact of ecological and demographic factors on the geographic spread of the virus. Moreover, the blood donor/recipient population studied here may be biased in respect to geographic distribution, age, gender or socio-economic status. However, we remain confident that the sample presented here is mostly representative of the population as sero-conversion follows the epidemic curve observed in Rio de Janeiro from surveillance data^22,41^. More comprehensive spatial and genomic sampling from unsampled regions are needed to unambiguously identify the drivers and hotspots of virus transmission^55–57^. Such quest could be achieved with analyses of large-scale blood banks combined with a real-time genomic surveillance network capable of discriminating between virus species and serotypes^58^. This would allow effective preventive measures to be designed and implemented efficiently in regions of greatest risk of importation and exportation at particular periods of elevated outbreak risk. Additional work could further evaluate intra-host diversity in symptomatic and asymptomatic patients.

In conclusion, joint epidemiological and genomic surveillance of blood banks as the potential to elucidate the drivers of arboviral transmission and to detect cryptic transmission of newly introduced mosquito-borne viral lineages and species, particularly into regions where generalist *Aedes* vectors circulate.

## Materials and Methods

### Patient cohort

The study protocol was approved by the Institutional Ethics Committee (Comissão de Ética em Pesquisa em Seres Humanos da Faculdade de Medicina da Universidade de São Paulo). During Feb-Jun 2012 all donors from the blood centers in Hemório (Rio de Janeiro municipality, Rio de Janeiro state) and Hemope (Recife municipality, Pernambuco state) were asked to participate in the study. An extra Plasma Preparation Tube (PPT; Becton Dickinson, Franklin Lakes, NJ) was collected from each consenting donor and recipient. Recipients who received components from a consenting donor were identified and invited to participate in the study. Once accepted, samples were collected 3–21 days after the index transfusion. All pre- and post-transfusion samples from enrolled recipients (*n* = 1,144), all donor samples from Rio de Janeiro (*n* = 15,866) and donor samples from Recife that were linked to enrolled recipients (*n* = 4,051) were tested by a transcription-mediated amplification (TMA) assay (Hologic/Grifols) that has a 50% detection limit of ~5 RNA copies/mL for each of the four DENV serotypes [19]. The study detected 149 DENV RNA + samples. Of those, 142 samples (119 donors and 23 recipients) had sufficient plasma volume to proceed to viral RNA extraction and whole genome PCR amplification (Table S1). Ethics committees in Brazil and the USA approved this study. The study design is described in detail in a previous study REDS-III^22^.

### Extraction, cDNA synthesis, PCR amplification and sequencing

All donor samples collected from individuals in RJc (*n* = 15,866), and the samples obtained from donors in Recife that were linked to enrolled recipients (*n* = 4,051) and 657 recipient samples were tested by a transcription mediated amplification assay (TMA; Hologic/Grifols). Plasma was centrifuged and viral RNA was extracted from supernatant with the Agencourt RNAdvance Tissue Kit (Beckman Coulter, Indianapolis IN, US) according to the manufacturer’s instructions. cDNA was synthesized with SuperScript III Reverse Transcriptase (Invitrogen, Carlsbad, CA, US), according to the manufacturer’s instructions using specific primer decamers (10 bp) complementary to the conserved region of 3’UTR of all dengue serotypes. Degenerate primers (described in Table S2) distributed along the complete genome were designed to amplify five overlapping fragments (2–4kb-long) covering the full genome (F1: 1–3790 bp, F2: 2884–4993 bp, F3: 4830–7330 bp, F4: 5982–8704 bp and F5: 7800–10649 bp, based on a DENV4 reference genome (Genbank NC_002640). PCR products were purified using the QIAquick PCR Purification Kit (QIAGEN, Stockach, Germany), following the manufacturer’s instructions and DNA concentrations were estimated using the Qubit dsDNA HS Assay Kit (Invitrogen, Carlsbad, CA, USA). The amplicons from a single viral genome were pooled at equimolar ratios. Prior to sequencing, amplicon mixtures (200ng) were sonicated to produce 200bp-long fragments using the Bioruptor (Diagenode Inc Denville, NJ, USA). A library was then built using the AB Library Builder System, Ion Xpress Barcode Adapters kit and Ion Plus Library Preparation kit (Invitrogen, Carlsbad, CA, USA), following the manufacturer’s instructions. Clonal amplification was performed using the Ion OneTouch, Ion PGM Template OT2 200 kit (Invitrogen, Carlsbad, CA, USA). Sequencing was performed using the PGM Instrument, the Ion PGM Sequencing 200 Kit v2 and the Ion 318 Chip. Datasets were then trimmed according the quality (99% coverage) and length (reads < 30 bp were removed) of each read using Geneious R7 software (Biomatters). The sequences of primers used for PCR were also removed. Full genome sequences were then reconstituted by mapping the reads to a reference sequence from GenBank using Geneious (majority base at each position)^59^. Sequences obtained were examined to ensure that the mapping to a reference sequence did not generate a biased consensus sequence.

### Dataset collation, sequence quality control and alignment

One hundred and fifty three DENV4 publicly-available and published near full-length genomes (>8000 bp) were retrieved in Feb 2016 using the ACNUC biological database system^60^. The genome sequences newly generated in this study were added to this global full-length DEN4 dataset. Multiple nucleotide sequence alignment was performed with MAFFTv.7^61^. After manually editing of the alignment^62^, a maximum likelihood (ML) tree (*n* = 244) was estimated using RAxML under general time reversible model with gamma-distributed rates ($GTR+{\rm{\Gamma }}$) nucleotide substitution model^63^. TempEst^64^ was used to inspect the molecular clock signal in the phylogeny and to identify potential contaminants. Three strains (N28351-Recife, N26684-Recife, N15054-Rio de Janeiro) were removed from subsequent analysis as they had an unusually high divergence compared to sequences collected within the same time frame. In addition, two sylvatic strains were removed from subsequent analysis to avoid introducing biases in evolutionary rate estimates. DENV types were classified using the Dengue, Zika and Chikungunya Viruses Typing Tool v.0.9 (http://bioafrica2.mrc.ac.za/rega-genotype-alpha/aedesviruses/typingtool/).

### Global molecular clock phylogeny of DENV4

Time-calibrated phylogenies were generated using the Bayesian Markov chain Monte Carlo (MCMC) framework implemented in BEAST v.1.8.4 package^65^. The BEAGLEv.2 library^66^ was used to enhance computational speed and a ML tree was used as a starting tree. For computational tractability, the best-fitting nucleotide substitution model was identified using path-sampling and stepping stone model selection (chain length 10 million steps and 100 path steps) using a subset of 40 isolates random chosen from the global dataset. The $GTR+{\rm{\Gamma }}$ outperformed both the HKY and the TN93 (not shown). To model changes in the effective population size over time, two non-parametric coalescent models were used, a Bayesian Skygrid model^67^ with 50 grid points and a Bayesian skyline model with 10 groups^68^. Markov chain Monte Carlo (MCMC) sampling was performed in duplicate for 250 million steps and convergence of the chains was inspected using Tracer v.1.6. After removing 10% burn-in, log and tree files were combined and a maximum clade credibility (MCC) tree was generated using TreeAnnotator^69^.

### Estimating epidemic spread using a birth-death skyline model

Dated phylogenies were reconstructed for the clade representing the DENV4 outbreak in Brazil (Fig. 2A). As expected, very little temporal signal was obtained using only the sequences belonging to the outbreak clade (root-to-tip regression analysis; r^2^ = 0.044; slope = 2.52 × 10^−3^ substitutions per site per year, s/s/y). Therefore, to perform time-calibrated phylogenetic reconstruction, we placed an informative lognormal prior on the TMRCA of the outbreak clade (mean 3.26 years before July 2012 and standard deviation 0.5) as obtained from analyses of the global dataset with strong temporal signal (Fig. 1F inset).

To estimate changes in epidemic spread through time, we used a Bayesian birth-death skyline plot model^70^ implemented in BEAST2^71^. In this model, each infection may transmit at a rate $\lambda$ and becomes noninfectious at a rate $\delta$. Upon becoming infected, each individual is sampled with a probability $s$ and included in the dataset. The model enables the piecewise estimation of *R*_e_ (the effective reproduction number of the outbreak), $\delta$ and $s$ through time. We assumed sampling proportion *s* to be constant over time. Relaxing this assumption to allow parameter *s* to be zero for the periods when no sequence data was available resulted in similar trends for *R*_e_ with wider Bayesian credible intervals (not shown). The rate $\delta$ was modelled using a lognormal prior with a mean of 14 days and a standard deviation of 0.5, which roughly corresponds to the sum of the intrinsic and extrinsic incubation period of dengue virus. The BDSKY analysis was run for 3 independent MCMC chains of 20 × 10^6^ steps, with parameters and trees being sampled once every 20000 steps. After removal of 10% burn in, sampled parameters were combined using LogCombiner^69^.

### Ento-epidemiological Susceptible-Exposed-Infected-Recovered model

To model the daily dynamics of DENV4-II infections in Rio de Janeiro, we used a Bayesian MCMC modelling approach based on framework previously used to investigate the first DENV1 outbreak in the Island of Madeira^35^. A time series for DENV4-II in Brazil was compiled from the number of positive cases from February to July 2012 within the RED-II cohort in Rio de Janeiro (Fig. S3). Weekly minimum temperature data for RJ was obtained from SEOMA/RJ, the Brazilian weather repository. In brief, the approach uses ordinary differential equations (ODE) to describe the dynamics of infection within the human and mosquito populations. The human population is assumed to be fully susceptible before the introduction of DENV4 and constant in size throughout the period of observation. Upon challenge with infectious mosquito bites, individuals enter the incubation phase, later becoming infectious, and finally recovering with life-long immunity (SI, Equations 1 to 5). For the dynamics of mosquito populations (SI, Equations 6 to 10), individuals were divided into two life-stages: aquatic and adult females. The adults are further divided into the epidemiologically relevant stages for DENV transmission: susceptible, incubating, and infectious for life.

Following the approach in^35^, the ODE model has 8 entomological parameters that are temperature-dependent (aquatic to adult transition rate, aquatic mortality rate, adult mortality rate, oviposition rate, incubation period, transmission probabilities per infectious bite, hatching success rate; Table S3). The MCMC approach estimates four unknown model parameters: the time of first human case, the aquatic carrying capacity, and two linear factors used to scale the effects of temperature on mosquito mortality and incubation period. Five other parameters are not estimated by the MCMC nor considered to be temperature-independent, for which we used literature-based estimates (mosquito biting rate, human incubation period, human infectious period, mosquito sex ratio, donor observation rate; Table S4). The Bayesian MCMC implementation and the ento-epidemiological SEIR model are described in detail in Supplementary Information, including the expressions for the effective and basic reproduction numbers of DENV^72,73^.

### Data availability

New sequence data have been deposited in Genbank under accession numbers (to be included). Genomic position, sequencing statistics, viral load and geographic location of the newly sequenced data is shown in Table S1. Code for the ODE model is available from the authors upon request.

### Ethical statement

All participants were informed of the study aims, and written informed consent was received from each patient before sample collection. The study protocol was approved by the Institutional Ethics Committee (Comissão de Ética em Pesquisa em Seres Humanos da Faculdade de Medicina da Universidade de São Paulo) and was in accordance with the Declaration of Helsinki for Human Research of 1974 (last modified in 2000). All experimental protocols were approved by the Institutional Ethics Committee (Comissão de Ética em Pesquisa em Seres Humanos da Faculdade de Medicina da Universidade de São Paulo).

### Biosafety

All protocols and procedures were conducted within the enhanced laboratory biosafety level 2 (ABSL-2) facility of Institute for Tropical Medicine, Sao Paulo University. The ABSL-2 facility consists of a laboratory in which all *in vitro* experimental work is carried out in class 3 biosafety cabinets, which are also negative pressurized (<−200 Pa). Although all experiments are conducted in closed class 3 cabinets and isolators, special personal protective equipment, including laboratory suits, gloves and FFP3 facemasks is used. Air released from the class 3 units is filtered by High Efficiency Particulate Air (HEPA) filters and then leaves via the facility ventilation system, again via HEPA filters. Only authorized personnel that have received the appropriate training can access the facility. The facility is secured by procedures recognized as appropriate by the institutional biosafety officers and facility management at Sao Paulo University and Brazilian National Technical Biosafety Commission (CTNBio).

## Electronic supplementary material

SUPPLEMENTARY INFO
