# Supplementary material for: Genomic and epidemiological characterisation of a dengue virus outbreak among blood donors in Brazil
Source: Sci Rep. 2017 Nov 9;7:15216. doi: 10.1038/s41598-017-15152-8 (PMC5680240; doi:10.1038/s41598-017-15152-8)
Supplement: Supplementary file 1 — SUPPLEMENTARY INFO [file 41598_2017_15152_MOESM1_ESM.pdf]

Supplementary Information

Genomic and epidemiological characterisation of a dengue virus outbreak among blood donors in Brazil.

Nuno R. Faria*‡, Antonio Charlys da Costa*‡, Jose Lourenço, Paula Loureiro, Maria Esther Lopes, Roberto Ribeiro, Cecilia Salete Alencar, Moritz U. G. Kraemer, Christian J. Villabona-Arenas, Chieh-Hsi Wu, Julien Thézé, Kamran Khan, Shannon E. Brent, Camila Romano, Eric Delwart, Brian Custer, Michael P. Busch, Oliver G. Pybus, Ester C. Sabino*, For the International Component of the NHLBI Recipient Epidemiology and Donor Evaluation Study-III (REDS-III).

‡ Contributed equally

* Correspondence: charlysbr@yahoo.com.br; nuno.faria@zoo.ox.ac.uk; sabinoec@usp.br

SI Materials and Methods…………………………………………………….. p. 1
SI Figures……………………………………………………….…………… p. 7
SI Tables………………………………………………………………..……. p. 12
SI References……………………………………………………………… p. 23

SI Materials and Methods

Ento-epidemiological dynamics model

The ordinary differential equations (ODE) model and the Markov-chain Monte Carlo (MCMC) approach herein used are based on the fitting framework previously proposed to study the 2012 dengue outbreak on the island of Madeira [1]. We have changed this framework to relax major modelling assumptions on the mosquito sex ratio and success of egg hatching, and have also transformed the original least squares based MCMC into a Bayesian MCMC.

The dynamics of infection within the human population are defined in Equations 1-5. In summary, the human population is assumed to have constant size (*N*) and to be fully susceptible before the introduction of a dengue virus lineage. Upon challenge with infectious mosquito bites $\lambda^{v \to h}$, individuals enter the incubation phase (E^h) with mean duration of $1/\gamma^h$ days, later becoming infectious (I^h) for $1/\sigma^h$ days and finally recovering (R^h) with life-long immunity.

$$\frac{dS^h}{dt} = -\lambda^{v \to h} \qquad (1)$$
$$\frac{dE^h}{dt} = \lambda^{v \to h} - \gamma^h E^h \qquad (2)$$
$$\frac{dI^h}{dt} = \gamma^h E^h - \sigma^h I^h \qquad (3)$$
$$\frac{dR^h}{dt} = \sigma^h I^h \qquad (4)$$
$$N = S^h + E^h + I^h + R^h \qquad (5)$$

For the dynamics of the mosquito population (Equations 6-10), individuals are divided into two pertinent life-stages: aquatic (eggs, larvae and pupae, *A*) and adult females *V* as in [2]. The adults are further divided into the epidemiologically relevant stages for dengue virus transmission: susceptible S^v incubating E^v for $1/\dot{\gamma}^v$ days and infectious I^v for life. The ˙ (dot) notation is here adopted to distinguish temperature-dependent entomological factors (further details in the following sections).

$$\frac{dA}{dt} = \dot{c} f \dot{\theta}_A^v \left(1 - \frac{A}{K}\right) V - (\dot{\epsilon}_A^v + \dot{\mu}_A^v) A \qquad (6)$$
$$\frac{dS^v}{dt} = \dot{c} f \dot{\theta}_A^v \left(1 - \frac{A}{K}\right) V - (\dot{\epsilon}_A^v + \dot{\mu}_A^v) A \qquad (7)$$
$$\frac{dE^v}{dt} = \lambda^{h \to v} - \dot{\gamma}^v E^v - \dot{\mu}_V^v E^v \qquad (8)$$
$$\frac{dI^v}{dt} = \dot{\gamma}^v E^v - \dot{\mu}_V^v E^v \qquad (9)$$
$$V = S^v + E^v + I^v \qquad (10)$$

Here, the coefficient $\dot{c}$ is the fraction of eggs hatching to larvae and *f* the resulting female proportion. For simplicity and lack of quantifications for local mosquito populations, it is assumed that the sex ratio remains at 1:1 (i.e. *f*=0.5). Moreover, $\dot{\epsilon}_A^v$ denotes the rate of transition from aquatic to adult stages, $\dot{\mu}_A^v$ the aquatic mortality, $\dot{\mu}_V^v$ the adult mortality, and $\dot{\theta}_A^v$ is the intrinsic oviposition rate. The logistic term $1 - \frac{A}{K}$ can be understood as the ecological capacity to receive eggs, scaled by a carrying capacity term *K*. From Equations 6-10, the mean number of viable female offspring produced by one female adult during its life-time, i.e. the basic offspring number *Q*, was derived (Equation 11). Most parameters defining *Q* are temperature-dependent, and for a fixed temperature T_0 expressions were derived for the expected population sizes

of each mosquito life-stage modelled, which are used to initialize the vector population (Equations 12-13).

$$Q = \frac{\dot{\epsilon}_A^v}{\dot{\epsilon}_A^v + \dot{\mu}_A^v} \frac{\dot{c} f \dot{\theta}^v}{\dot{\mu}_V^v} \quad (11)$$

$$A(T_0) = K\left(1 - \frac{1}{Q(T_0)}\right) \quad (12)$$

$$V(T_0) = K\left(1 - \frac{1}{Q(T_0)}\right) \frac{\epsilon_A^v(T_0)}{\mu_V^v(T_0)} \quad (13)$$

Viral Transmission

In respect to the *infected host-type* being considered, the vector-to-human ($\lambda^{v \to h}$) and human-to-vector ($\lambda^{h \to v}$) incidence rates are assumed to be, respectively, density-dependent and frequency-dependent (equations 14-15). Here, a is the biting rate and $\dot{\theta}^{v \to h}$ and $\dot{\phi}^{h \to v}$ are the vector-to-human and human-to-vector transmission probabilities per bite. Conceptually, this implies that (i) an increase in the density of infectious vectors should directly raise the risk of infection to a single human, while (ii) an increase in the frequency of infected humans raises the risk of infection to a mosquito biting at a fixed rate. The basic reproductive number (R_0) is defined similarly to previous modelling approaches (Equation 16) [3, 4], and the effective reproduction number (R_e) is derived *de novo* (Equation 17). For R_e, the relevance of herd-immunity is clear from the factor $\left(\frac{S^h}{N^h}\right)\left(\frac{S^v}{N^h}\right)$, which modulates the difference to R_0. When the population is fully susceptible, $\frac{S^h}{N^h} = 1$ and $\left(\frac{S^v}{N^h}\right)$=$\left(\frac{V}{N^h}\right) = M$ and hence $R_0 = R_e$, otherwise $R_0 \neq R_e$.

$$\lambda^{v \to h} = \left(\frac{a \dot{\phi}^{v \to h} I^v S^h}{N^h}\right) \propto I^v \quad (14)$$

$$\lambda^{h \to v} = \left(\frac{a \dot{\phi}^{h \to v} I^h S^v}{N^h}\right) \propto \frac{I^h}{N^h} \quad (15)$$

$$\dot{R}_0 = \frac{(V/N^h)(S^v/N^h) a^2 \dot{\phi}^{h \to v} \dot{\gamma}^v}{\dot{\mu}_V^v \sigma^h (\dot{\gamma}^v + \dot{\mu}_V^v)} \quad (16)$$

$$\dot{R}_e = \frac{(S/N^h)(S^v/N^h) a^2 \dot{\phi}^{v \to h} \dot{\phi}^{h \to v} \dot{\gamma}^v}{\dot{\mu}_V^v \sigma^h (\dot{\gamma}^v + \dot{\mu}_V^v)} \quad (17)$$

1.3. Temperature-Dependent Parameters

For each of the temperature-dependent entomological parameters (Table S3), temperature-dependent polynomial expressions are found *de novo* or taken from previous studies, in which temperature is considered in both Celsius (T) and Kelvin T_k, and the universal gas constant R is taken in $cal\ deg^{-1} mol^{-1}$. The expressions for the mortality ($\dot{\mu}_A^v, \dot{\mu}_V^v$), aquatic to adult ($\dot{\epsilon}_A^v$) and ovipositon ($\dot{\theta}^v$) rates are taken from the study by Yang and colleagues under temperature-controlled experiments on

populations of *Aedes aegypti* (Equations 17-20) [5]. The relationship between the extrinsic incubation period ($1/\gamma_V^v$) and temperature were taken from the formulation used in Focks et al. [6], which assumes that replication is determined by a single rate-controlling enzyme (Equation 21) [6-8]. The probabilities of transmission per mosquito bite ($\dot{\phi}^{h\to v}$, $\dot{\phi}^{v\to h}$) are modelled here (Equations 22-23) as in Lambrechts and colleagues [9]. Finally, the relationship between temperature and the fraction of eggs that successfully hatch ($\dot{c}$) is estimated *de novo* (equation 24) by fitting a third-degree polynomial to *Aedes aegypti* and *Aedes albopictus* empirical data described in SI Fig S1.

$$\dot{\epsilon}_A^v = \dot{\epsilon}_A^v\,(T) = 0.131 - 0.05723T + 0.01164T^2 - \ 0.001341T^3 + 0.00008723T^4 - 0.000003017T^5 + 5.153\times 10^{-8}T^6 - 3.42 \times 10^{-10}T^7 \tag{18}$$

$$\dot{\mu}_A^v = \ \dot{\mu}_A^v(T) = 2.13 - 0.3797\mathrm{T} + \ 0.02457T^2 - 0.0006778T^3 + 0.000006794T^4 \tag{19}$$

$$\dot{\mu}_V^v = \dot{\mu}_V^v(T) = 0.8692 - 0.1599\mathrm{T} + 0.01116T^2 - 0.0003408T^3 + 0.000003809T^4 \tag{20}$$

$$\dot{\theta}_V^v = \theta_V^v(T) = -5.4 + 1.8T - 0.2124T^2 + 0.01015T^3 - 0.00001515T^4 \tag{21}$$

$$\dot{\gamma}_V^v = \gamma_V^v(T) = \frac{0.003359\frac{T_k}{298}\times exp\left(\frac{15000}{R}\left(\frac{1}{298}-\frac{1}{T_k}\right)\right)}{1+exp\left(\frac{6.203\times 10^{21}}{R}\left(\frac{1}{-2.176\times 10^{30}}-\frac{1}{T_k}\right)\right)} \tag{22}$$

$$\dot{\phi}^{h\to v} = \phi^{h\to v}(T) = 0.001044T \ \times \ (T - 12.286) \times \ (32.3461 - T)^{1/2} \tag{23}$$

$$\dot{\phi}^{v\to h} = \phi^{v\to h}(T) = 0.0729T - 0.97 \tag{24}$$

$$\dot{c} = c(T) = (-184.8 \ + 27.94T - \ 0.9254T^2 + 0.009226T^3)/100 \tag{25}$$

1.4. Constant Parameters

The framework described above has only 5 fixed parameters that are neither temperature-dependent nor estimated in the MCMC approach (Table S4). Amongst these, the blood donor observation rate (ς) represents the composed proportion of

blood donors and observed cases in Rio de Janeiro for the time period modelled. For simplicity, we assume the virologically confirmed rate in donors to be ≈ 1, such that (ς) can be interpreted as the proportion of individuals in Rio de Janeiro that are blood donors.

1.5. Markov Chain Monte Carlo Fitting Approach

For the fitting process, the MCMC algorithm by Lourenco et al. is here altered to a Bayesian approach by formalising a likelihood and parameter priors [1]. For this, the jumping distributions of each parameter were kept as Gaussian (symmetric), effectively retaining a random walk Metropolis-Hastings kernel. We define our acceptance probability α of a parameter set Θ, given model ODE output y as:

$$\alpha = min\left\{1, \frac{\pi(y|\Theta^*)\pi(\Theta^*)}{\pi(y|\Theta^0)\pi(\Theta^0)}\right\} \qquad (26)$$

where Θ^* and Θ^0 are the proposed and accepted parameter sets (respectively), and $\pi(y|\Theta^*)$ and $\pi(y|\Theta^0)$ are the likelihoods of the ODE output representing the epidemic data, given each parameter set. We assume uniform priors to all estimated parameters, and the likelihoods to be the product of the conditional Poisson probabilities of each epidemic data and ODE point:

$$\pi(y|\Theta) = \prod_{i=1}^{N}[Pr\{y_i = d_i\}] \qquad (27)$$

To address MCMC convergence, we quantified $\sqrt{\hat{R}}$, the Gelman-Rubin statistic (which compares the variance between and within M independent MCMC chains) [10]. This statistic is expected to approximate 1 when M independent chains have converged to the same stationary distributions. Values significantly larger than 1, for instance, indicate that the between-chain variance is greater that the within-chain variance, highlighting that the MCMC may need more time to converge or tuning of jump parameters is required [10]. For calculation details please refer see [1].

1.6. Fitted Parameters

With the MCMC approach described above, all combinations of the *open* parameters in the ODE system that most likely represent the outbreak are explored (Table S5). In summary, the MCMC estimates distributions for: (1) the carrying capacity *K*, used to indirectly estimate the number of adult mosquitoes per human; (2) time point of the first case t_0, assumed to be in a human; (3) a linear coefficient η that scales the effect of temperature on aquatic and adult mortality rates; and (4) a linear coefficient α that scales the effect of temperature on the extrinsic incubation period. By introducing the

linear coefficients η and α, the relative effect of temperature variation on mortality and incubation is not changed *per se*, but instead the baselines are allowed to be different from the laboratory ideal conditions used by Yang et al. in laboratory experiments [5]. For a discussion on possible biological factors that may justify η and α we refer to the original description of the method [1] and to [11] .

SI Figures

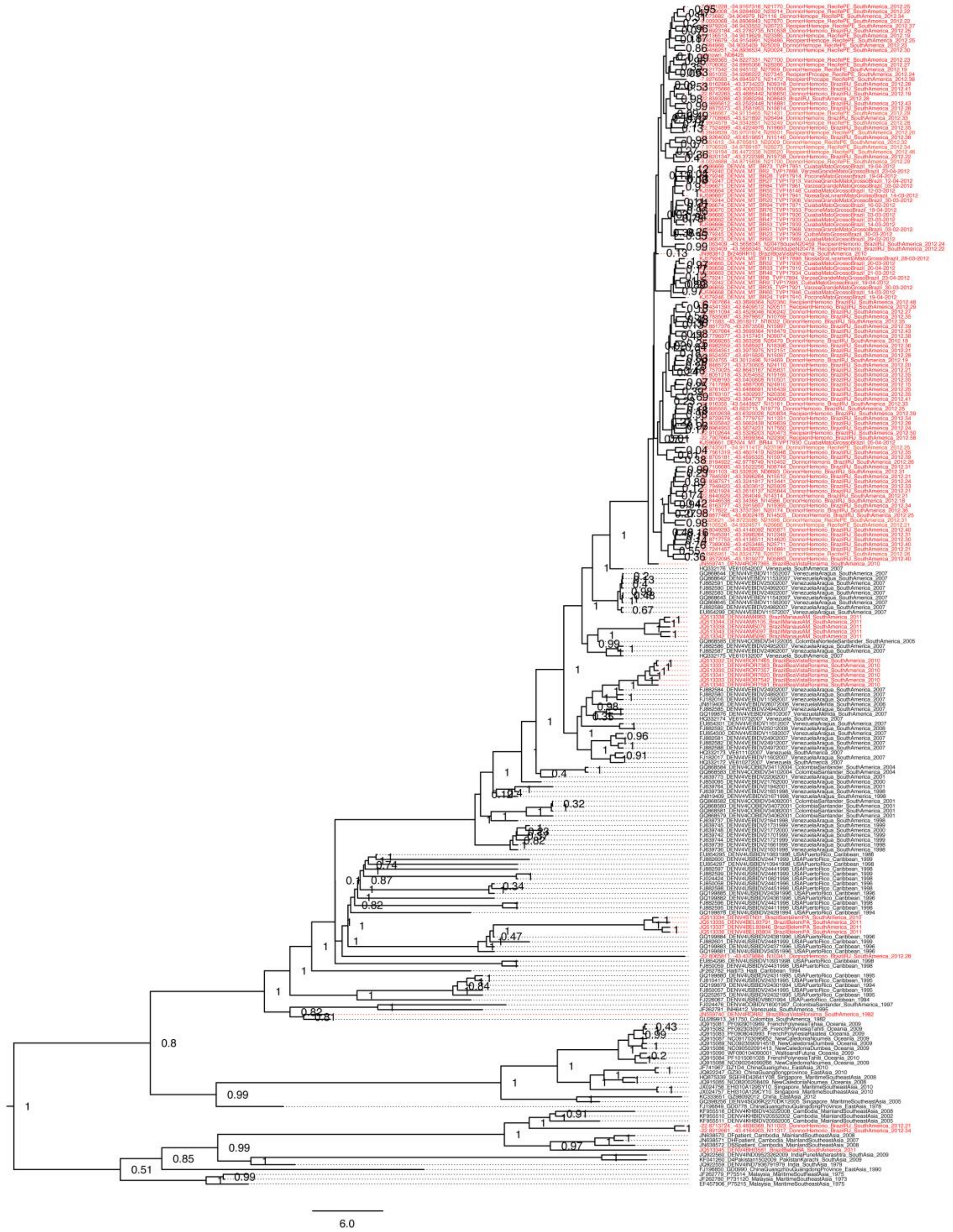

SI Fig 1. Dated phylogeny of dengue virus serotype 4. Maximum likelihood phylogenetic of a global sample (n=244) of dengue virus serotype 4 complete and near complete genomes (>8000 bp). Sequences in red depict isolates sampled in Brazil. Numbers indicate clade posterior probability support.

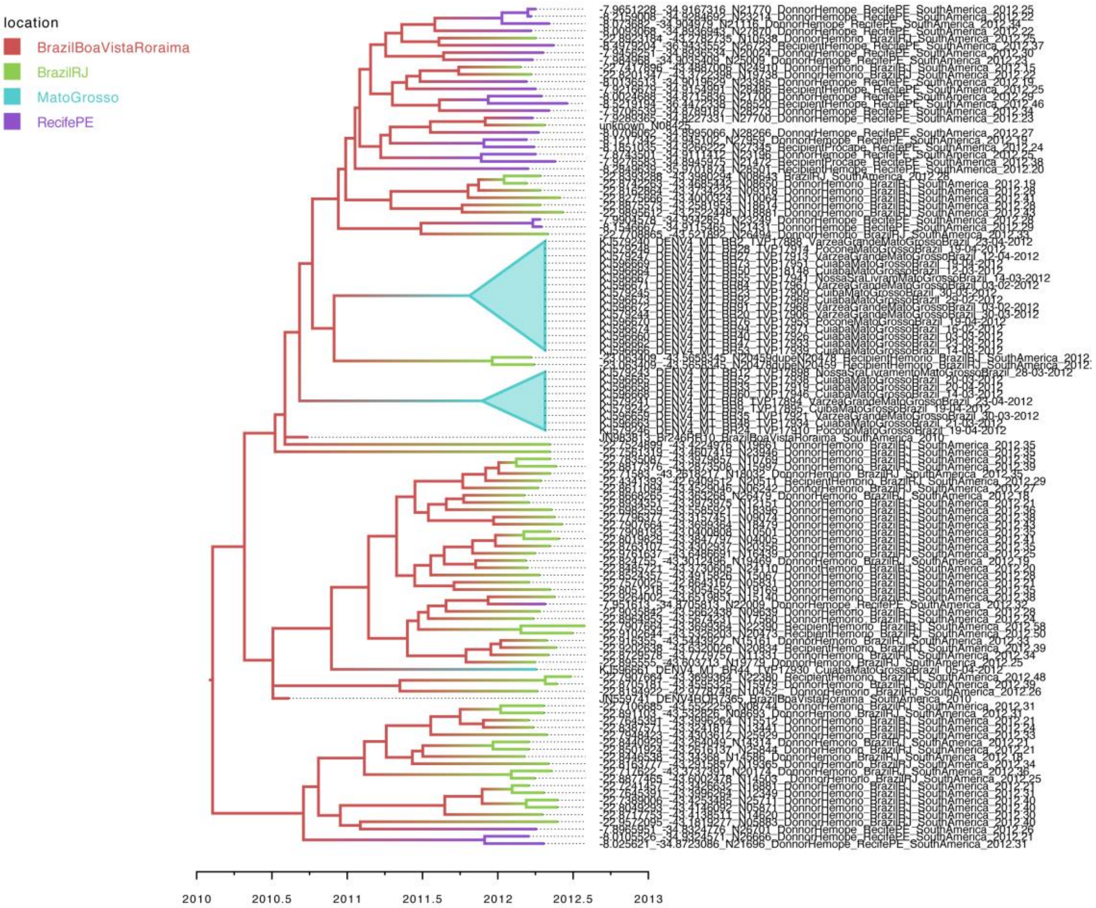

SI Fig 2. Phylogeographic tree of the Brazilian dengue virus serotype 4 genotype II. Colours indicate most probable locations as inferred using an asymmetric discrete trait model (see Materials and Methods).

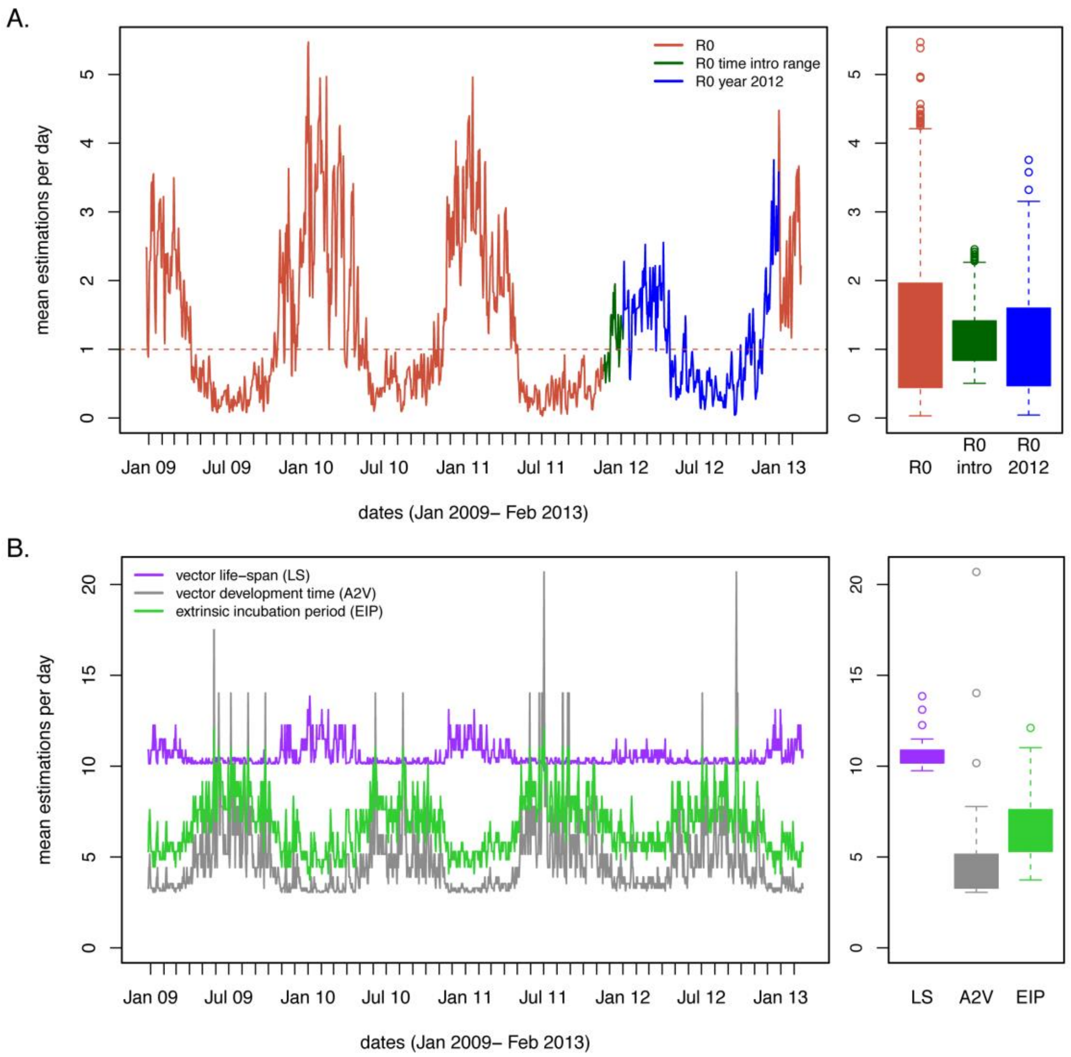

SI Fig 3. Epidemiological and entomological dynamics of dengue virus outbreak in Rio de Janeiro. Panel A. Daily estimates obtained using the ento-epidemiological model for the effective reproductive number. The box on the right-hand side shows the estimated R0 over the entire period of observation, for the time period where viral introduction is estimated to have occurred (green) and for the year 2012 alone (blue). **Panel B**. The green line corresponds to the lifespan of the *Aedes* mosquitoes, while the purple line corresponds to the vector's extrinsic incubation period.

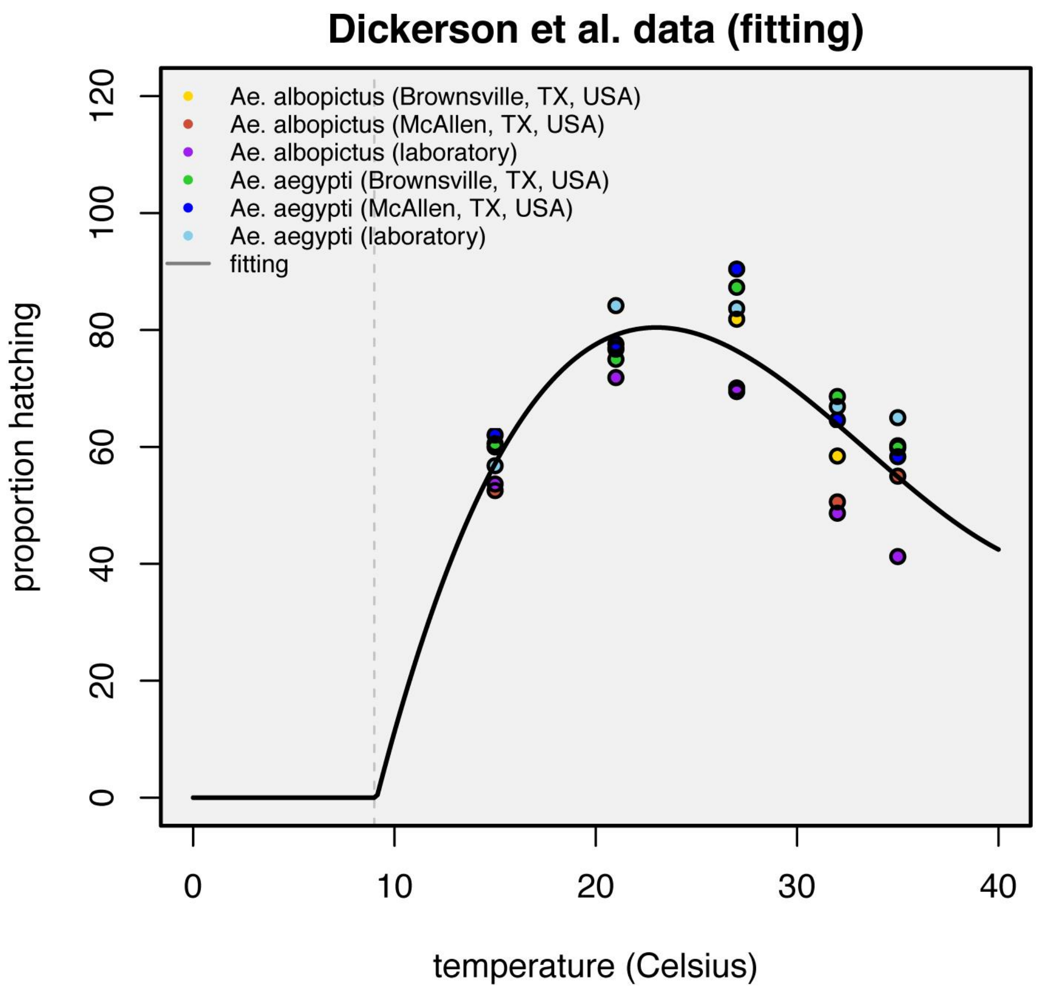

SI Fig 4. Relationship between temperature and egg hatching success. Empirical data on *Aedes aegypti* and *Aedes Albopictus* egg hatching success ($\dot{c}$) is taken from [9]. Data includes measurements of hatching for 5 different temperatures above 15 Celsius, including 2 wild and 1 laboratory populations for each of the vector-species. Fitting implemented with a third-degree polynomial in R (which can be found in the Results section of the main text). When modeling, negative proportions below 10 Celsius are manually corrected to zero (left of shaded grey line).

SI Fig. 5.

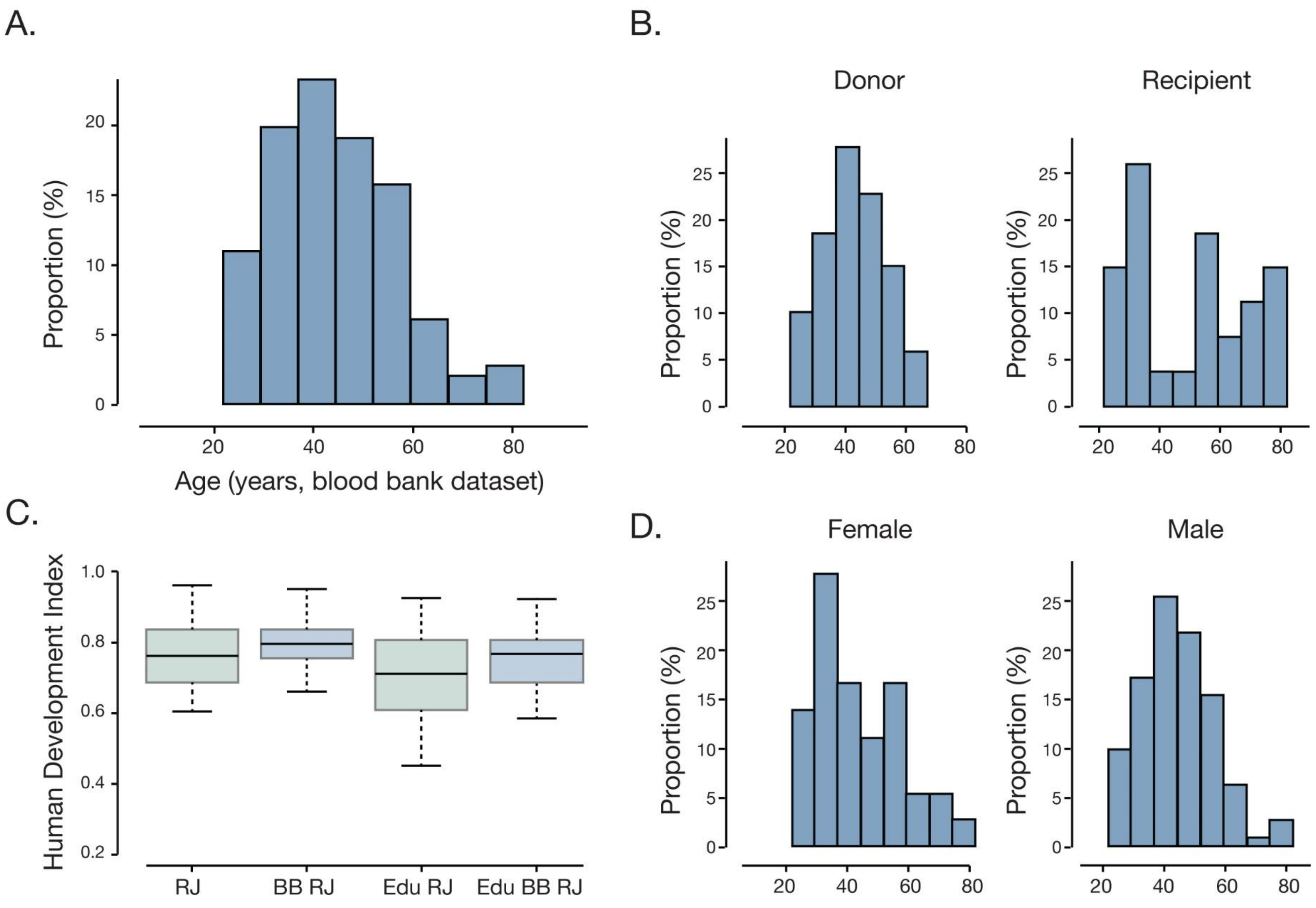
A.
Proportion (%)
0
5
10
15
20
20
40
60
80
Age (years, blood bank dataset)
B.
Donor
Recipient
Proportion (%)
0
5
10
15
20
25
20
40
60
80
C.
Human Development Index
0.2
0.4
0.6
0.8
1.0
RJ
BB RJ
Edu RJ
Edu BB RJ
D.
Female
Male

SI Tables

Table S1. Genomic position, sequencing statistics, viral load and geographic location of the samples used in this study.

ID	Position of the fragment in relation to complete genome*					Length of consensus sequence	Number of reads	Coverage	Viral load estimated by qPCR	City	State
	F01 1-3790	F02 2884-4993	F03 4830-7330	F04 5982-8704	F05 7800-10649						
DEN007761	+	+	+	+	+	10602	17701275	1670	1244000	Rio de Janeiro	RJ
N05871	+	+	+	+	Fail	7728	16744636	2167	127100	Nilópolis	RJ
N09074	+	+	+	+	+	10617	18225813	1717	197400	Duque de Caxias	RJ
N09318	+	+	+	+	+	10621	19868891	1871	513000	Rio de Janeiro	RJ
N10769	+	+	+	+	+	10149	23026731	2269	161800	São Joao de Meriti	RJ
N11331	+	+	+	+	+	10196	21898535	2148	347000	Rio de Janeiro	RJ
N12151	+	+	+	+	+	10173	21560038	2119	32000	Rio de Janeiro	RJ
N14314	+	+	+	+	+	10400	24044682	2312	10560000	Rio de Janeiro	RJ
N15161	+	+	+	+	+	10165	32297884	3177	595000	Rio de Janeiro	RJ
N15512	+	+	+	+	+	10616	33694398	3174	759000	Belford Roxo	RJ
N15997	+	+	+	+	+	10600	28561888	2695	406000	Rio de Janeiro	RJ
N18032	+	+	+	+	+	10600	36009922	3397	25300000	Duque de Caxias	RJ
N18479	+	+	+	+	+	10173	25839532	2540	212000	São Joao de Meriti	RJ
N18614	+	+	+	+	Fail	8800	55980944	6361	107200	Rio de Janeiro	RJ
N18881	+	+	+	+	+	10600	26798856	2528	165500	Rio de Janeiro	RJ
N19169	+	+	+	+	+	10013	37079159	3703	19150	Rio de Janeiro	RJ
N19365	+	+	+	+	+	10500	38006533	3620	15210000	Rio de Janeiro	RJ
N19661	+	+	+	+	+	10558	39666938	3757	113400	Nova Iguaçu	RJ
N20174	+	+	+	+	+	10141	17864024	1762	131300	Belford Roxo	RJ
N21431	+	+	+	+	+	10602	35818508	3378	49300	Recife	PE
N23249	+	+	+	+	+	10600	32765414	3091	126300	Recife	PE
N24110	+	+	+	+	+	10120	30887951	3052	1061000	Rio de Janeiro	RJ
N28266	+	+	+	+	+	10503	26065920	2482	49500	Recife	PE
N08425	Fail	+	+	+	Fail	5405	38083532	7046	Not performed	Duque de Caxias	RJ

N08744	+	+	+	+	Fail	7010	17865236	2549	1227	Queimadas	RJ
N08643	+	+	+	+	+	10100	21857285	2164	25200	Rio de Janeiro	RJ
N08650	+	+	+	+	+	10100	12684914	1256	7910	Rio de Janeiro	RJ
N10341	+	+	+	+	Fail	7291	73710209	10110	3660	Mesquita	RJ
N04005	+	+	+	Fail	Fail	6722	22712293	3379	1987	São Joao de Meriti	RJ
N09639	+	+	+	+	Fail	7670	28078280	3661	3410	Rio de Janeiro	RJ
N11023	+	+	+	+	+	10660	16744636	1571	2500	Rio de Janeiro	RJ
N14586	+	+		Fail	Fail	4200	25736669	6128	822	Rio de Janeiro	RJ
N14620	Fail	+	+	Fail	Fail	4635	14329751	3092	11350	Rio de Janeiro	RJ
N10452	Fail	+	Fail	Fail	Fail	2883	9831331	3410	4510	São Gonçalo	RJ
N19738	Fail	+	Fail	Fail	Fail	2285	15417715	6747	9000	Rio de Janeiro	RJ
N20356	Fail	+	+	Fail	Fail	4142	18178554	4389	5750	Rio de Janeiro	RJ
N21696	+	+	+	+	+	10053	13603379	1353	48	Olinda	PE
N23249	+	+	+	+	+	10451	24500166	2344	Not performed	Recife	PE
N10064	+	+	+	+	+	10572	16765871	1586	833	Rio de Janeiro	RJ
N18396	Fail	+	Fail	Fail	Fail	2248	25033980	11136	590	Queimados	RJ
N20024	+	+	+	+	+	10550	27951145	2649	863	Paulista	PE
N23946	+	+	+	+	+	10509	32273318	3071	40700	Nova Iguaçu	RJ
N25009	+	+	+	+	+	10470	12460586	1190	1200	Olinda	PE
N28351	Fail	+	Fail	Fail	Fail	2105	36432296	17308	200	Olinda	PE
N26666_DR3	+	+	+	+	+	10450	12651318	1211	1542	Recife	PE
N27372R_DR3[§]	Fail								100	Recife	PE
N06242_DR5	Fail								36	Rio de Janeiro	RJ
N20511R_B_DR5[§]	+	+	+	+	Fail	9185	29287022	3189	64700	Cachoeiras de Macacu	RJ
N22221R_B_DR5[§]	Fail								24	Cachoeiras de Macacu	RJ
N22234R_B_DR5[§]	Fail								Not performed	Cachoeiras de Macacu	RJ
N26479_DR4	+	+	+	+	+	10400	28510152	2741	84400	Rio de Janeiro	RJ
N20463R_DR4[§]	Fail								100	Belford Roxo	RJ

N27959_DR2	+	+	+	+	Fail	8704	21211153	2437	2720	Recife	PE
N27345R_C_DR 2§	Fail								200	Jaboatão dos Guararapes	PE
N27350R_C_DR 2§	Fail								200	Jaboatão dos Guararapes	PE
N20458R_DR6§	Fail								12	Petrópolis	RJ
N15979_DR6	+	+	+	+	Fail	7509	12441076	1657	336	Rio de Janeiro	RJ
N21495R_DR1§	Fail								48	Jaboatão dos Guararapes	PE
N23385_DR1	+	+	Fail	Fail	Fail	4953	24266541	4899	200	Recife	PE
N20459R_A§	+	+	+	+	+	10610	52153491	4916	241000	Rio de Janeiro	RJ
N20478R_A§	+	+	Fail	Fail	Fail	5818	68018491	11691	11300	Rio de Janeiro	RJ
N20473R§	+	+	+	+	+	10500	14286937	1361	60900	Rio de Janeiro	RJ
N22380R§	Fail	Fail	Fail	+	Fail	2795	17959071	6425	1597	São João de Meriti	RJ
N22390R§	+	+	+	+	Fail	8504	51076136	6006	490	São João de Meriti	RJ
N20834R§	+	+	Fail	Fail	Fail	5340	48164549	9020	200	Rio de Janeiro	RJ
N26723R§	+	+	+	Fail	Fail	7040	42620119	6054	396	Pedra	PE
N21472R§	+	+	+	+	+	10560	23342368	2210	25400	Paulista	PE
N28501R§	+	+	+	+	+	10400	15464682	1487	353	Caruaru	PE
N28486R§	+	+	Fail	Fail	Fail	5083	46682005	9184	203	Abreu e Lima	PE
N28520R§	+	+	+	+	Fail	7685	27408884	3567	214	São Bento do Uma	PE
N23571R§	Fail								Not performed	Belo Jardim	PE
N28132R§	Fail								200	Brejão	PE
N22222R§	Fail								12	Rio de Janeiro	RJ
N20841R§	Fail								100	Rio de Janeiro	RJ
N23513R§	Fail								24	Recife	PE
N22356R§	Fail								48	Nova Iguaçu	RJ
N22250R§	Fail								36	Rio de Janeiro	RJ
N25929	+	+	+	+	Fail	8392	39166310	4667	78600	Mesquita	RJ
N27870	+	+	+	+	Fail	8677	35624996	4106	4010	Recife	RJ
N08693	+	+	+	Fail	Fail	7618	53945772	7081	69000	Rio de Janeiro	RJ

N05831	+	+	+	+	Fail	8836	51039102	5776	83000	Itaboraí	RJ
N26684	+	Fail	Fail	Fail	Fail	4775	48010747	10055	12	Recife	RJ
N15067	+	+	+	+	+	10500	18976839	1807	794000	Rio de Janeiro	RJ
N13441	+	+	+	+	Fail	8811	42661026	4842	48800	Rio de Janeiro	RJ
N15140	+	+	+	+	Fail	8385	36771884	4385	7390	Rio de Janeiro	RJ
N14503	+	+	+	+	+	10480	41264904	3937	6120000	Rio de Janeiro	RJ
N05883	+	+	+	+	Fail	8005	25484912	3184	33100	Rio de Janeiro	RJ
N16881	+	+	Fail	Fail	Fail	4990	40667219	8150	12260	Belford Roxo	RJ
N19779	+	+	+	+	+	10607	20995408	1979	49000	Rio de Janeiro	RJ
N19469	Fail	+	+	Fail	Fail	4445	26151599	5883	48200	Rio de Janeiro	RJ
N21700	+	+	Fail	Fail	Fail	4995	20048411	4014	339	Olinda	PE
N27700	+	+	+	Fail	Fail	6522	31624778	4849	5050	Paulista	PE
N10501	Fail	+	Fail	Fail	Fail	2144	29912290	13952	893	São Gonçalo	RJ
N12349	+	Fail	Fail	Fail	Fail	1215	25790573	21227	200	Belford Roxo	RJ
N15054	+	Fail	Fail	Fail	Fail	1218	21170704	17382	12	Mesquita	RJ
N11317	+	Fail	Fail	Fail	Fail	1802	27307768	15154	215	Rio de Janeiro	RJ
N26494	Fail	Fail	Fail	Fail	+	2799	31792918	11359	200	Nova Iguaçu	RJ
N25711	+	Fail	Fail	Fail	Fail	1675	13496804	8058	356	Belford Roxo	RJ
N10538	Fail	+	+	+	Fail	6378	17335596	2718	392	Rio de Janeiro	RJ
N16439	Fail	+	Fail	Fail	Fail	2229	18857541	8460	200	Rio de Janeiro	RJ
N24910	+	+	Fail	Fail	Fail	5443	16070048	2952	16340	Nova Iguaçu	RJ
N25844	+	+	+	Fail	Fail	6364	32103759	5045	57800	Rio de Janeiro	RJ
N26701	Fail	+	Fail	Fail	Fail	1385	22903689	16537	200	Paulista	PE
N23196	+	Fail	Fail	Fail	Fail	Fail	32397868		200	Igarassu	PE
N28273	+	+	Fail	Fail	Fail	4717	21661814	4592	200	Paulista	PE
N23214	+	+	+	+	+	10397	27474973	2643	852	Jaboatão dos Guararapes	PE
N21770	+	+	+	+	+	10179	21439718	2106	200	Recife	PE
N17560	+	+	+	+	+	10154	44970348	4429	225000	Rio de Janeiro	RJ
N22009	Fail	+	Fail	Fail	Fail	2385	23075034	9675	200	Paulista	PE

N21979	Fail				200	Pesqueira	PE
N20875	Fail				3100	Rio de Janeiro	RJ
N06359	Fail				Not performed	Rio de Janeiro	RJ
N14886	Fail				Not performed	Rio de Janeiro	RJ
N14629	Fail				685	Rio de Janeiro	RJ
N15110	Fail				1116	Belford Roxo	RJ
N26330	Fail				Not performed	Nova Iguaçu	RJ
N25987	Fail				24	Recife	PE
N20612	Fail				Not performed	Recife	PE
N25759	Fail				12	Rio de Janeiro	RJ
N24195	Fail				100	Mesquita	RJ
N06081	Fail				200	Rio de Janeiro	RJ
N11138	Fail				Not performed	Rio de Janeiro	RJ
N08874	Fail				100	Rio de Janeiro	RJ
N06513	Fail				100	Rio de Janeiro	RJ
N15472	Fail				200	Rio de Janeiro	RJ
N17254	Fail				48	São João de Meriti	RJ
N11462	Fail				200	Rio de Janeiro	RJ
N19423	Fail				300	Nova Iguaçu	RJ
N17954	Fail				24	Rio de Janeiro	RJ
N22458	Fail				48	Paulista	PE
N27440	Fail				Not performed	Abreu e Lima	PE
N27964	Fail				200	Jaboatão dos Guararapes	PE
N28249	Fail				200	Recife	PE
N27535	Fail				100	Recife	PE
N25336	Fail				12	Nilópolis	RJ
N26890	Fail				36	Goiana	PE
N21116	Fail				48	Recife	PE
N12090	Fail				Not performed	Nova Iguaçu	RJ
N12707	Fail				Not performed	Rio de Janeiro	RJ
N13343	Fail				Not performed	São João de Meriti	RJ
N18730	Fail				Not performed	Nova Iguaçu	RJ
N12333	Fail				36	Rio de Janeiro	RJ

N13148	Fail				Not performed	Nova Iguaçu	RJ
N16526	Fail				244	Mesquita	RJ
N10770	Fail				12	São João de Meriti	RJ
N14169	Fail				36	São Gonçalo	RJ
N25596	Fail				100	Rio de Janeiro	RJ
N25078	Fail				200	Rio de Janeiro	RJ
N17018	Not enough volume				Not performed	Nova Iguaçu	RJ

Notes:

* Genomic position in relation to DENV4 reference strain (Accession Number: NC_002640).

§ Sample IDs followed by letter "R" indicate that individuals were blood receptors, while the remainder are blood donos. Sample IDs that have A, B or C in the end represent sequences from the same individual, with differente collection dates. Sample IDs with DR followed by a number between 1 and 6, represent donor and recipiente pairs. Six individuals were confirmed by serological tests to have received blood-derived products with dengue vírus.

Table S2. Description of the primers used in cDNA reactions, amplification and Sanger sequencing of the viral genome. The positions are based on DENV reference strain (Genbank No. NC_002640). The sequences were modified to cover all known genotypes.

Name	Sequence 5' - 3'	Position
D1-4_RT_R	GRWTCAACRC	10640 - 10649
D1-4_RT_F	AGTTGTTAGKCTACGTGGAC	1 - 20
D4_RT_R	AGAACCTGTTGGATCAACAAC	10629 - 10649
D4_01F	GATGAGGGAAGATGGGGAGTTGTTAGTCTGTGTGGACCGAC	-17 - 24
D4_01R	TGGTTGATCTRAYTCCACAGAC	2583 - 2604
D4_02F	GGRAAAAATCCYAAACATGTCC	4830 - 4851
D4_02R	ACYTGCCCTAATTGYTTTTCAAA	7309 - 7331
D4_03F	AAAGAYATYCCGCAGTGGGAACC	9630 - 9652
D4_03R	TTTGTCGGTCTGGGGGGGTATAGAACCTGTTGGATCAACAAC	10629 - 10670
D4_04F	CTCCRTGYAAAGTCCCCATAGAGA	1930 - 1953
D4_05F	CYCACTGGACAGAAGCAAAGATG	5981 - 6003
D4_06F	AGRACAGCTGCTGGGATCATGA	7225 - 7246
D4_04R	TGTGRAARTGGTGGGAGCAAAA	9687 - 9708
D4_05R	TTCCTTGAGTTCGTRCCRATCC	2332 - 2353
D4_06R	CATTATGCCCTCGTTRAGRGGCC	4138 - 4160
D4_07R	TCTTTGCTTCTGTCCAGTGRGC	5980 - 6001
D4_07F	TCGGAAGCTTGCTTAACACA	40 - 59
D4_08F	CTCCGTGTAAAGTCCCCATAGAGA	1930 - 1953
D4_09F	AGRACAGCTGCTGGGATCATGA	7225 - 7246
D4_10F	AACAAATGCACYCTYATTGCCA	534 - 555
D4_11F	TTTGAACTGACYAAGACAACAGC	1065 - 1087
D4_12F	GYTCCATTGGCAAGATGTTTGAG	2125 - 2147
D4_13F	GGGTTGTGTGGTGTCATGGA	2429 - 2448
D4_14F	AGGTGGAAGACTATGGATTTGG	2884 - 2905
D4_15F	GGAGATTAGGCCCTTGAGTGAAAA	3422 - 3445
D4_16F	TGGCTTGGAGGACCATTATGG	3964 - 3984
D4_17F	TCAGGTCTCTACCCCTTGGC	4452 - 4471
D4_18F	GAAATGGGAGAGGCAGCAGC	5437 - 5456
D4_19F	CACTGGACAGAAGCAAARATG	5983 - 6003
D4_20F	TGGCTAGTGGCTTRCTYTGG	6647 - 6666
D4_21F	CTTGGYTGYGGGAGAGGAGG	7800 - 7819
D4_22F	GCACAAAGAAACCTGGCAYTATGA	8423 - 8446
D4_23F	CCGAGCAATCTGGTAYATGTGG	8975 - 8996
D4_24F	AAAGACATYCCGCAGTGGGA	9630 - 9649
D4_25F	GACAAGACTCCAGTCCATTCGTG	10041 - 10063
D4_26F	GCCATGCGCCACGGAAGCTGT	10381 - 10401
D4_08R	TCATRAGGGGTTCRCCRTCTCT	456 - 477
D4_09R	GTYTCCARTCCCATTCCTGA	735 - 754
D4_10R	TGTGRAARTGGTGGGAGCAAAA	9687 - 9708
D4_11R	ACYTGCCCTAATTGCTTTTCAAA	7309 - 7331

D4_12R	CCATYCTCTCTTTRTRATTC	1631 - 1650
D4_13R	AAYTTTCCYGARCACATCGT	1836 - 1855
D4_14R	CAYGAGACYCCYCCAAACAT	2277 - 2296
D4_15R	TTTCCAGYCTNGTGGTTGATC	2596 - 2616
D4_16R	GTRGTGGTYCTCAAAGATGG	3309 - 3328
D4_17R	TGAYTTRACCATGTTCTCTTC	3447 - 3467
D4_18R	ACCATTAGYGCTGTCTCTCT	3771 - 3790
D4_19R	GAGTGCTGTTATTTCTACCCA	4050 - 4070
D4_20R	CAAACCCACAGCCATTATGCC	4152 - 4172
D4_21R	GTTATGTCTGCCATTTCATCCC	4315 - 4336
D4_22R	CCAGTGAAGCACCATTCCCG	6202 - 6221
D4_23R	ATTGATTGTCTTGTGGGGTCC	6758 - 6778
D4_24R	TTGGTRAACYACTCCATTTCC	4974 - 4994
D4_25R	TGGGTGTCTCCATTGTGGACTG	1363 - 1384
D4_26R	TTTCCCRAAYAACCCTCTTTGC	4601 - 4622
D4_27R	TCAACTGGATHACTYTCTTTCCCG	5667 - 5680
D4_28R	TTGTCTTTCCRGCTCCGGGGT	5105 - 5125
D4_29R	CATCTGTTCYGTGATCAGTTCYTCA	9209 - 9233
D4_30R	ARCCAATTGGCTGTCGTGGT	8685 - 8704
D4_31R	GARTTCCTGGAYARCGGGCA	8184 - 8203
D4_32R	CCATCTYTYAGGGCAGACTTGG	7685 - 7706
D4_33R	TTCCGATCAGRTTCCTGACCTG	10170 - 10191

Table S3. Temperature-dependent parameters. All expressions except the egg hatching success (see Fig. S4) were taken from published work.

Notation	Description	Ref.
$\dot{\epsilon}_A^v = \dot{\epsilon}_A^v\,(T)$	Transition rate from aquatic to adult mosquito life-stages	[5]
$\dot{\mu}_A^v = \dot{\mu}_A^v(T)$	Mortality rate of aquatic mosquito life-stage	[5]
$\dot{\mu}_V^v = \dot{\mu}_V^v(T)$	Mortality rate of adult mosquito life-stage	[5]
$\dot{\theta}_V^v = \theta_V^v(T)$	Intrinsic oviposition rate of adult mosquito life-stage	[5]
$\dot{\gamma}_V^v = \gamma_V^v(T)$	Extrinsic incubation period of adult mosquito life-stage	[6]
$\dot{\phi}^{h\rightarrow v} = \phi^{h\rightarrow v}(T)$	Human-to-vector probability of transmission per infectious bite	[9]
$\dot{\phi}^{v\rightarrow h} = \phi^{v\rightarrow h}(T)$	Vector-to-human probability of transmission per infectious bite	[9]
$\dot{c} = c(T)$	Egg hatching success	Fig. S4

Table S4. Constant parameters. Parameters that are fixed in the ODE system.

Notation	Value	Description	Ref.
a	0.33 per day	Mosquito biting rate	[12]
$1/\gamma^h$	2 days	Human latency period	[13, 14]
$1/\sigma^h$	4 days	Human infectious period	[15, 16]
f	0.5	Proportion of female (sex ratio)	[17, 18]
ς	0.05	Blood donor observation rate	-

Table S5. Estimated parameters. Free parameters used by the Bayesian MCMC approach to fit the data.

Notation	Description	Ranges
t_0	Time point of first case (in a human)	(∞, ∞)
K	Aquatic carrying capacity	$(0, \infty)$
η	Multiplicative (linear) factor for mosquito mortality	$(0, \infty)$
α	Multiplicative (linear) factor for extrinsic incubation period	$(0, \infty)$

References

1. Lourenco, J. & Recker, M. The 2012 Madeira dengue outbreak: epidemiological determinants and future epidemic potential. *PLoS neglected tropical diseases* **8**, e3083, doi:10.1371/journal.pntd.0003083 (2014).
2. Yang, H. M., Macoris, M. L., Galvani, K. C., Andrighetti, M. T. & Wanderley, D. M. Assessing the effects of temperature on dengue transmission. *Epidemiology and infection* **137**, 1179-1187, doi:10.1017/S0950268809002052 (2009).
3. Lourenco, J. & Recker, M. Natural, persistent oscillations in a spatial multi-strain disease system with application to dengue. *PLoS computational biology* **9**, e1003308, doi:10.1371/journal.pcbi.1003308 (2013).
4. Wearing, H. J. & Rohani, P. Ecological and immunological determinants of dengue epidemics. *Proceedings of the National Academy of Sciences of the United States of America* **103**, 11802-11807, doi:10.1073/pnas.0602960103 (2006).
5. Yang, H. M., Macoris, M. L., Galvani, K. C., Andrighetti, M. T. & Wanderley, D. M. Assessing the effects of temperature on the population of Aedes aegypti, the vector of dengue. *Epidemiology and infection* **137**, 1188-1202, doi:10.1017/S0950268809002040 (2009).
6. Focks, D. A., Daniels, E., Haile, D. G. & Keesling, J. E. A simulation model of the epidemiology of urban dengue fever: literature analysis, model development, preliminary validation, and samples of simulation results. *Am J Trop Med Hyg* **53**, 489-506 (1995).
7. Otero, M., Solari, H. G. & Schweigmann, N. A stochastic population dynamics model for Aedes aegypti: formulation and application to a city with temperate climate. *Bull Math Biol* **68**, 1945-1974, doi:10.1007/s11538-006-9067-y (2006).
8. Schoolfield, R. M., Sharpe, P. J. & Magnuson, C. E. Non-linear regression of biological temperature-dependent rate models based on absolute reaction-rate theory. *J Theor Biol* **88**, 719-731 (1981).
9. Lambrechts, L. *et al.* Impact of daily temperature fluctuations on dengue virus transmission by Aedes aegypti. *Proceedings of the National Academy of Sciences of the United States of America* **108**, 7460-7465, doi:10.1073/pnas.1101377108 (2011).
10. Gelman, A. a. R., DB Inference from iterative simulation using multiple sequences. *Statistical Science* **7** (1992).
11. Brady, O. J. *et al.* Modelling adult Aedes aegypti and Aedes albopictus survival at different temperatures in laboratory and field settings. *Parasit Vectors* **6**, 351, doi:10.1186/1756-3305-6-351 (2013).
12. Trpis, M. & Hausermann, W. Dispersal and other population parameters of Aedes aegypti in an African village and their possible significance in epidemiology of vector-borne diseases. *Am J Trop Med Hyg* **35**, 1263-1279 (1986).
13. Chan, M. & Johansson, M. A. The incubation periods of Dengue viruses. *PloS one* **7**, e50972, doi:10.1371/journal.pone.0050972 (2012).
14. Halstead, S. B. Dengue. *Lancet* **370**, 1644-1652, doi:10.1016/S0140-6736(07)61687-0 (2007).

15 Gubler, D. J., Suharyono, W., Tan, R., Abidin, M. & Sie, A. Viraemia in patients with naturally acquired dengue infection. *Bull World Health Organ* **59**, 623-630 (1981).
16 Vaughn, D. W. *et al.* Dengue viremia titer, antibody response pattern, and virus serotype correlate with disease severity. *J Infect Dis* **181**, 2-9, doi:10.1086/315215 (2000).
17 Lounibos, L. P. & Escher, R. L. Sex ratios of mosquitoes from long-term censuses of Florida tree holes. *J Am Mosq Control Assoc* **24**, 11-15, doi:10.2987/5656.1 (2008).
18 Mohammed, A. & Chadee, D. D. Effects of different temperature regimens on the development of Aedes aegypti (L.) (Diptera: Culicidae) mosquitoes. *Acta Trop* **119**, 38-43, doi:10.1016/j.actatropica.2011.04.004 (2011).
